# Precise metabolomics identifies glycolysis-related pyruvate kinase M activity as regulator of the S-phase-specific radiation response in triple-negative breast cancer cells

**DOI:** 10.1186/s12964-026-02803-5

**Published:** 2026-03-12

**Authors:** Rocío Matesanz-Sánchez, Sandra Classen, Kanstantsin Siniuk, Mirko Peitzsch, Tiago Alves, Helmut Pospiech, Kerstin Borgmann, Nils Cordes

**Affiliations:** 1 OncoRay—National Center for Radiation Research in Oncology, Faculty of Medicine Carl Gustav Carus, Technische Universität Dresden, Dresden, 01307 Germany; 2 Department of Radiotherapy & Radiation Oncology, Hubertus Wald Tumor Center, University Cancer Center Hamburg, University Medical Center Hamburg-Eppendorf, Hamburg, 20246 Germany; 3 Project Group Biochemistry, Leibniz Institute on Aging - Fritz Lipmann Institute, Jena, 07745 Germany; 4 Institute of Clinical Chemistry and Laboratory Medicine, University Hospital Carl Gustav Carus, Technische Universität, Dresden, 01307 Germany; 5 Institute for Medical Informatics and Biometry (IMB), Technische Universität Dresden, 01307 Dresden, Germany; 6 Faculty of Biochemistry and Molecular Medicine, University of Oulu, Oulu, 90014 Finland; 7 Research laboratory of the Clinic for Gynecology and Obstetrics, University Hospital Düsseldorf, Düsseldorf, Germany; 8 Institute of Radiooncology— OncoRay, Helmholtz-Zentrum Dresden-Rossendorf (HZDR), Dresden, 01328 Germany; 9 German Cancer Consortium (DKTK), German Cancer Research Center (DKFZ), Heidelberg, 69120 Germany; 10 Department of Radiotherapy and Radiation Oncology, University Hospital Carl Gustav Carus, Technische Universität Dresden, Dresden, 01307 Germany

**Keywords:** Triple-negative breast cancer, Metabolomics, Pyruvate Kinase M, Radiosensitization, DNA replication stress

## Abstract

Metabolic reprogramming is known to critically contribute to therapy response in cancer, including the resistance of triple-negative breast cancer (TNBC) patients to conventional radio/(chemo)therapy. Our study, therefore, characterized the metabolic response of TNBC cell models to irradiation using untargeted metabolomics. We identified radiation-induced metabolic changes by harnessing a metabolite-gene interaction network comprising 44 genes. Nine genes with significant radiosensitizing potential in TNBC cells were identified by RNAi-screening. Among the druggable genes, high expression of pyruvate kinase M (PKM) was associated with worse overall survival in the METABRIC-TNBC cohort. Pharmacological PKM inhibition led to radiosensitization in TNBC cell models, which was attributed to S-phase related metabolic changes. Selective disruption of the S-phase by PKM inhibition led to both reduced DNA synthesis and increased replication stress, as evidenced by increased DNA damage at active replication forks. This resulted in prolonged cell cycle arrest after irradiation. Metabolic profiling upon PKM depletion and irradiation revealed two positively PKM-associated gene clusters, predominantly involved in the glycolysis pathway. Each cluster was functionally distinct, one shared the PKM-associated effects in survival reduction, while the other correlated with its radiosensitization. High expression of the first cluster was significantly correlated with worse overall survival, whereas high expression of the second cluster, consisting of PKM, ENO1, GAPDH and GPI, predicted poor response to radiotherapy in the METABRIC-TNBC cohort. Thus, our study suggests that glycolytic-associated metabolic reprogramming upon irradiation plays a major role in radioresistance of TNBC. Analysis of our identified glycolysis-related gene cluster in patients could predict the response to radiotherapy, and targeting PKM and the related glycolytic pathway holds promise for radiosensitization in TNBC patients.

**Supplementary Information:**

The online version contains supplementary material available at 10.1186/s12964-026-02803-5.

## Background

Triple-negative breast cancer (TNBC) is a highly aggressive subtype of breast cancer. It is characterized by the absence of estrogen and progesterone receptors and lack of human epidermal growth factor receptor 2 (HER2) amplification. Compared to other breast cancer subtypes, TNBC often presents with a less favorable prognosis [1], partially due to the development of resistance to conventional radio- and chemotherapy [2]. This highlights the need for new therapeutic options. One strategy for the discovery of novel therapeutic targets is the unraveling of the cancer metabolome, with untargeted metabolomics playing a significant role in identifying biomarkers and therapeutic targets [3–6].

Metabolism rewiring, a hallmark in almost all cancer types, is clearly associated with radiation resistance in breast cancer, displaying changes in glycolysis, the tricarboxylic acid (TCA) cycle, and fatty acid metabolism [6–8]. These metabolic adaptations ensure survival and proliferation while intricately regulating various cellular functions, including gene expression via epigenetic modifications [9]. Particularly, glucose metabolism is one of the most investigated metabolic pathways in the context of radiation resistance [10]. One of the key enzymes of glycolysis is the pyruvate kinase M (PKM), the rate-limiting enzyme catalyzing the final step in the synthesis of pyruvate [11]. Despite the fact that a variety of human malignancies highly express the PKM2 isoform, both PKM2 and PKM1 isoforms play a critical role in cancer cell proliferation and confer selective growth advantages by regulating gene expression in the nucleus, orchestrating cell cycle progression, maintaining redox homeodynamics, and modulating cell metabolism [12–14]. In TNBC specifically, high activity of PKM has been correlated to worse prognosis, enhanced tumor growth, and chemotherapy resistance [15]. Due to these diverse functions, it is not surprising that PKM is also centrally involved in providing metabolic precursors for ensuring adequate DNA replication [16, 17] and DNA damage response (DDR), which contribute to radiation resistance [18]. Rare reports provide initial evidence for the interlinkage of the metabolome and genome stability by providing metabolic intermediates essential for DNA damage repair. This applies to both processes important for DNA double-strand break repair, homologous recombination (HR) and non-homologous end joining [19].

To address radiation-induced metabolic changes in TNBC cell models in more depth, we performed metabolome analyses and hypothesized that metabolic alterations influence the regulatory framework for the energy-demanding DNA replication and DDR processes, thereby modulating radiosensitivity. Our study identified radiation-induced metabolic changes and generated a metabolite-gene interaction network. Among these, druggable genes linked to radiosensitization were identified, with high expression of PKM showing a significant association with worse overall survival (OS) in the METABRIC-TNBC patient cohort. Functional studies combining PKM inhibition and irradiation revealed PKM-dependent alterations in clonogenic survival, metabolism, DDR, DNA replication, and cell cycling. Intriguingly, the observed changes in metabolic pathways and enzymes after PKM inhibition revealed specific associated gene clusters whose expression levels appear to be prognostic for survival and radiosensitivity of TNBC patients. Notably, high expression of these genes was significantly associated with poorer OS and a weaker response to radiotherapy in the METABRIC-TNBC patient cohort.

## Methods

### Cell lines

Human TNBC cell lines: MDA-MB-231, purchased from American Type Tissue Culture Collection (ATCC, HTB-26) (Manassas, VA, USA), Cal-85-1 (ACC 440), Cal-51 (ACC 302), both purchased from DSMZ, MDA-MB-468, Hs 578T, and BT-549 (kindly provided by S. Joosse; University Medical Center Hamburg-Eppendorf, Hamburg, Germany). All cell models, excluding Cal-51, were cultured in conditioned Dulbecco’s Modified Eagle’s Medium (DMEM, PAN-Biotech, Aidenbach, Germany) supplemented with 1 mM sodium pyruvate (PAN-Biotech), 2 mM L-glutamine (Sigma Aldrich), and 10% fetal bovine serum (PAN-Biotech). Cal-51 cells were cultured without sodium pyruvate supplementation. Cells were maintained at 37 °C and 5% or 8.5% CO_2_, regularly tested negative for mycoplasma contamination, and authenticated through STR DNA profiling.

### Radiation exposure

Cells underwent irradiation at room temperature with 6 Gy of γ-irradiation facilitated by a Caesium-137 source (Gammacell 40 (GC40) Irradiator, Nordion), or X-ray radiation applying 200 kV X-rays filtered with 0.8 mm Be + 0.5 mm Cu (Yxlon Y.TU 320; Yxlon, Hamburg, Germany or RS225 Gulmay Medical, Surrey, UK). The dose rate during irradiation was approximately 1.3 Gy/min at 20 mA–1.2 Gy/min at 15 mA. To ensure accuracy, the absorbed dose was measured using a Duplex dosimeter (PTW, Freiburg, Germany).

### Antibodies

Primary antibodies: 53BP1 (#NB-100-305, Novus Biologicals, CO, USA, 1:500), CDK2 (#SC-163, Santa Cruz Biotechnology, Dallas, TX, USA, 1:1000), pCDK2 [T14] (#ab68265, abcam, Cambridge, UK, 1:1000), Cyclin A (#SC-751, Santa Cruz Biotechnology, 1:400), Cyclin D1 (#CC12, Calbiochem/Merck Millipore, MA, USA, 1:1000), H2AX (#7631, Cell Signaling Technology, Danvers, MA, USA, 1:1000), PKM (#10078-2-AP, Proteintech, Planegg-Martinsried, Germany, 1:1000), RAD51 (#PC130, Calbiochem/Merck Millipore, 1:500), β-actin (#4970S; Cell Signaling Technology, 1:10000), γH2AX [Ser139] (#05-636 clone JBW301, Merck Millipore, 1:250). Secondary antibodies for immunofluorescence: Alexa Fluor 488 goat anti-rabbit IgG (#4412S, Cell Signaling, 1:600) and Alexa Flour 647 goat anti-mouse IgG (#4410S, Cell Signaling, 1:500). Secondary antibodies for western blot: IRDYE 680 anti-mouse IgG, IRDYE 800 anti-mouse IgG, IRDYE 680 anti-rabbit IgG, or IRDYE 800 anti-rabbit IgG purchased from LI-COR Biosciences (Lincoln, NE, USA) (1:7500) and EasyBlot anti Mouse IgG (HRP) (#GTX221667-01), EasyBlot anti Rabbit IgG (HRP) (#GTX221666-01) purchased from GeneTex (Irvine, CA, USA).

### Small interfering RNA knockdown

ON-TARGETplus siRNA library was purchased from Dharmacon™-Horizon Discovery (Cambridge, UK). This library included four specific siRNA sequences targeting each gene: ACLY, AKT1, ALDOB, ALG13, ALG5, B3GALNT1, B3GALT6, B4GALNT1, B4GALT1, B4GALT4, B4GALT7, CDC42, CDS1, CDS2, CEPT1, CHSY1, CMPK1, CTPS2, CYP27A1, DCK, ENO1, GAPDH, GCNT1, GCNT2, GPI, GYG1, GYS2, HAS1, HAS3, HRAS, IDH2, MGAT5, NBN, NME1, NME6, NME7, OGDH, PFKL, PFKP, PIGA, PKLR, PKM, RAB7A, RAC1, RHOA, RHOB, RRM2, SLC2A1, UGCG, UGT1A1, UGT1A7, UGT8, UXS1, VEGFA and three non-targeting controls. For siRNA transfection, cells were seeded 18 h prior incubation with Opti-MEM (Thermo Fisher Scientific, Waltham, MA, USA) including 9.1 nM siRNA and 5 µl lipofectamine RNAiMAX reagent (Invitrogen, Waltham, MA, USA) for 24 h, as published [20].

### Colony formation assay

For assessing clonogenicity, single cells were seeded in 6-well plates as published [21] and after 16–24 h, cells were irradiated with 6 Gy of X-rays and subsequently cultured for a cell line-specific duration until visible colonies were formed. For the determination of the IC_50_ of osalmid (#S3641, Selleckchem, Houston, USA), lonafarnib (#1457, Sigma-Aldrich), compound 3 K (#S8616, Selleckchem), and rhosin (#5003, Tocris Bioscience, Bristol, UK), components were added 16 h after seeding. For combination treatments, the IC_50_ of the components was added 6 h prior to X-ray irradiation with 2, 4–6 Gy. Colonies were fixed using 70–80% EtOH and stained with Coomassie blue (Merck Millipore) or 1% crystal violet (Sigma-Aldrich). Colonies with ≥ 50 cells were counted under a stereomicroscope (Carl Zeiss Microscopy GmbH, Oberkochen, Germany).

### Metabolome sample preparation

For untargeted metabolomics, 5 × 10^5^ MDA-MB-231 cells were seeded in a 10 cm dish. After 48 h, cells were irradiated with 6 Gy of γ-irradiation. Cells were washed twice in cold PBS 1 h post irradiation, followed by metabolite extraction using 2 mL of -80 C cold quenching buffer (80/20 MeOH/H2O; Optima™ LC/MS (Fisher Bioreagents, Pittsburgh, Pennsylvania, USA)/Molecular Biology Grade H_2_0 (Fisher Bioreagents)) and a 20-minute incubation at -80 °C. Cells were scraped into the same extraction buffer within an ice bath. The resulting cell suspension underwent two sequential centrifugations (14000 x g, 4 °C for 10 min), and the resulting supernatant was stored at -80 °C until processing, which involved drying the sample extract supernatants using vacuum-assisted centrifugation. Targeted metabolite profiling in MDA-MB-231 cells growing in 10-cm dishes was conducted 48 h after siRNA transfection and 1 h after exposure to 6 Gy X-ray irradiation. Cells underwent two cold PBS washes followed by cell extraction with the addition of a quenching solution (20% Methanol HPLC grade, 0.1% Formic acid, 3mM NaF, 100 μm EDTA). Cellular extracts were frozen and freeze-dried (Labconco) before preparation for MS analysis.

### Untargeted metabolomics

Metabolite profiling was conducted using liquid chromatography high-resolution tandem mass spectrometry (LC-HR-MS/MS) following an untargeted metabolomics approach as previously published [20]. The instrumentation comprised an ultra-performance liquid chromatography system (Aquity I-class; Waters GmbH, Eschborn, Germany) connected to a quadrupole- time-of-flight mass spectrometer (QToF) equipped with an ion mobility spectrometer (IMS) (VION IMS QToF; Waters GmbH, Eschborn, Germany). For analysis, the sample extract residue was reconstituted in 200 µL aqueous acetonitrile (with 10% water) containing 0.1% formic acid and transferred into autosampler glass vials. Additionally, a pool containing 20 µL from each sample was separately prepared and randomly analyzed multiple times during the analysis process. Chromatographic separation utilized hydrophilic interaction chromatography (HILIC) employing a BEH Amide column (2.1 × 100 mm, 1.7 μm; Waters GmbH, Eschborn, Germany) maintained at 45 °C. The mobile phase consisted of a gradient of water including 0.1% formic acid as a mobile phase A and acetonitrile including 0.1% formic acid as a mobile phase B (acetonitrile including 0.1% formic acid). The sample injection commenced with a mixture of 1%/99% A/B at a flow rate of 0.4 mL/min, followed by a linear increase in mobile phase A to 5% within 2 min, reaching the 99% reached at 7.5 min. After an additional 0.5 min, the initial conditions were restored within 0.3 min, followed by column equilibration at starting conditions for 1.7 min. For ionization, positive and negative electrospray ionization (ESI) combined with high-definition data acquisition scan mode (HDMSE) was employed, including ion mobility screening, determination of metabolite-specific collision cross-section (CCS) values, accurate mass, and respective fragment ion mass screenings. The observed mass range was between mass-to-charge ratios between 50 and 1000 Da. The total scan time was set to 0.3 s with 40% applying collision energy of 6 eV (low energy) and the remaining 60% ramping collision energy from 15 to 50 eV or 10 to 80 eV (high energy) in positive or negative ESI mode. The instrument parameters for positive ESI mode were set to 1 kV and 40 V for capillary voltage and sample cone voltage, with source and desolvation temperatures set at 120 °C and 550 °C, respectively. In negative ESI mode, parameters were set to 1.5 kV and 40 V, with source and desolvation temperatures at 120 °C and 450 °C, respectively. Nitrogen was used as both cone gas and desolvation gas, with flow rates of 50 L/h and 1000 L/h, respectively, in positive and negative ESI. For mass correction, repeated injections of Leucine-Enkephalin were employed. Data acquisition and raw data processing were conducted using the Waters Unifi software package 2.1.2. Subsequently, Unifi export files derived from positive and negative ESI screenings were imported into the Progenesis QI software (Nonlinear Dynamics, Newcastle upon Tyne, UK) for further data processing. This processing included peak picking with auto threshold and chromatographic alignment using an automatically chosen reference sample, signal deconvolution, normalization of the data, and experimental design setup. For metabolite identification, features from untargeted metabolomics screenings were compared with the Human Metabolome Data Base (HMDB) [22] using the Progenesis Metascope plugin, applying a precursor mass accuracy of ≤ 10 ppm and a theoretical fragment mass accuracy of ≤ 10 ppm. Additionally, features were compared to in-house data achieved by injecting metabolite standards in a neat solution, including allowed deviations of retention time and CCS of 0.3 min and 3%, respectively. Metabolic features without metabolite annotation were excluded, and the remaining features, along with their signal intensities and respective identifications were exported to Microsoft Excel for further data processing.

### Targeted metabolomics: metabolites from TCA cycle and glycolysis

Freeze-dried extracts were resuspended in water containing D4-Taurine and used as an external loading control (Cambridge Isotope Laboratories). Samples were maintained in an autosampler (Shimadzu SIL-40 C XR) at 4 °C before being injected into a Hypercarb column (100 × 4.6 mm, 3 μm, ThermoFisher). Chromatographic separation of metabolites was achieved over 10 min using an isocratic gradient (7mM ammonium formate (Sigma Aldrich), 1.5% acetonitrile (Biosolve Chimie, ULC/MS grade), 10 µM EDTA (Sigma Aldrich)) at a flow rate of 0.8 mL/min (Shimadzu LC-40D XR). The oven temperature was set at 45 °C (Shimadzu CTO-40 C). Ionization by electrospray occurred in a SCIEX 7500 QTRAP with the following source parameters: temperature 550 °C, CAD = 9, CUR = 55, GS1 = 50, GS2 = 75 and IS=-4500. The mass Q1/Q3 pairs used for each metabolite are listed in Supplementary Table S1. Peak identity was confirmed based on the retention time of a standard mixture, and MS data were integrated using El-Maven software (v0.12.0) and processed as fold change.

### Total protein extraction and western blotting

Cells were lysed 48 h after transfection or 24 h after PKMi (IC_50_) treatment, with 1 x RIPA lysis buffer (50 mM Tris HCl (pH 7.4, AppliChem, Darmstadt, Germany), 150 mM NaCl (Roth, Karlsruhe, Germany), 1% Nonidet-P40 (Sigma-Aldrich), 0.25% sodium deoxycholate,150 mM NaCl, 1 mM EDTA, 1 mM NaVO4, 2 mM NaF (all Sigma-Aldrich), Complete™ Protease Inhibitor Cocktail (Roche, Basel, Switzerland) or Halt™ Protease and Phosphatase Inhibitor Cocktail (100X) (ThermoFisher). Protein concentration measurements were performed using BCA assay kit (Thermo Fisher Scientific). Following SDS-PAGE using 4%–15% gradient gel (Bio-Rad Laboratories, Feldkirchen, Germany), proteins were transferred onto nitrocellulose membranes (GE Healthcare, Chicago, IL, USA) and detected by incubating the membranes with specific primary antibodies and horseradish peroxidase-conjugated or IRDYE-conjugated secondary antibodies as previously described [20, 23]. Protein bands were detected with an ECL Prime Western Blotting Detection Reagent (GE Healthcare) using a Fusion FX (Vilber Lourmat GmbH, Eberhardzell, Germany) or Odyssey CLx Infrared Imaging System (LI-COR Biosciences). Protein bands were normalized to β-actin.

### Proliferation assay

Fifty thousand cells were seeded per T25 flasks, cultured in growth media and allowed to attach overnight before treatment. PKM inhibitor (IC_50_) was added 6 h before X-ray irradiation. The cell number was determined via Beckman Coulter cell counter (Life Science, Krefeld, Germany) at each day after treatment over a time course of seven days.

### Cell viability

Cell viability was assessed by measuring ATP levels with CellTiter-Glo Luminescent Cell Viability Assay (Promega, Walldorf, Germany). One day after siRNA transfection, cells were seeded in a cell model-dependent density (500–1000 cells per well) in a white wall 96-well plate (Thermo Fisher Scientific). The following day, cells were either irradiated with 6 Gy or left unirradiated. After 5–7 days, ATP levels were determined following the manufacturer´s instructions. In brief, CellTiter-Glo reagent was mixed in a 1:1 ratio with the culture medium for 2 min. on an orbital shaker and incubated for 10 min. at room temperature. Luminescence was recorded during an integration time of 0.5 s using microplate reader SpectraMax^®^ iD3 (Molecular Devices, San Jose, CA, USA).

### Immunofluorescence for DNA damage foci

One hundred thousand cells were seeded in 12-well plates on culture slides (diameter 15 mm). After 24 h, cells were treated with 6 Gy X-ray irradiation, PKM inhibitor (IC_50_), or PKM inhibitor (IC_50_) plus 6 Gy applied after 6 h. Cells were permeabilized with 0.05% TritonX (Sigma-Aldrich) and fixed with 4% paraformaldehyde (Morphisto GmbH, Offenbach am Main, Germany) 24 h after treatment, and blocked overnight in 3% BSA (Sigma-Aldrich) at 4 °C. Foci number and colocalization were detected using primary antibodies against RAD51, γH2AX, and 53BP1. Nuclei were stained with DAPI. The quantification was performed automatically by the Aklides^®^ system (MediPan, Blankenfelde-Mahlow, Germany). Pan-nuclear γH2AX signal was counted manually from the MediPan pictures. A minimum of 100 cells per treatment and slide were quantified [20, 23, 24].

For the 5-Ethynyl-2’-Deoxyuridine (EdU) staining, cells were pulse-labelled with the base analogue EdU (#C10339, Invitrogen, 1:1000) for 20 min before fixation with 4% paraformaldehyde. EdU was stained according to the manufacturer’s protocol (#C10339, Invitrogen, 1:250). Cells were examined using the Axioplan 2 fluorescence microscope (Carl Zeiss Microscopy, Jena, Germany). RAD51 foci in EdU-negative or -positive cells and corrected total cell fluorescence (CTCF) were quantified using ImageJ software. A minimum of 100 cells per treatment and slide were analyzed [25].

### Cell cycle analysis

One hundred thousand cells were seeded per T25 flask and 24 h after seeding treatments were performed: 6 Gy irradiation, PKM inhibitor (IC_50_), or PKM inhibitor (IC_50_) plus 6 Gy irradiation applied after 6 h. Flow cytometry analysis was performed using a MACSQuant10 with MACSQuantify Software 2.11 (Miltenyi Biotec, Bergisch Gladbach, Germany) [25]. The proportion of cells in the respective cell cycle phases was calculated using ModFit LT™ 3.2 software (Verity Software House, Topsham, ME, USA). Analysis was performed as described before [23].

### DNA fiber assay

Exponentially growing cells were treated for 6 h with the PKM inhibitor (IC_50_), followed by pulse labeling with 25 µM 5-chloro-2′-deoxyuridine (CldU; Sigma-Aldrich) and 250 µM 5-iodo-2′-deoxyuridine (IdU; Sigma-Aldrich) for 30 min. The irradiated cells, received 6 Gy X-ray irradiation in between the CldU and IdU label, were harvested, and DNA fiber spreads were prepared and stained as described previously [24]. DNA fibers were examined using an Axioplan 2 fluorescence microscope (Carl Zeiss Microscopy). CldU and IdU tract lengths were measured using ImageJ software.

### Public database analysis

For in silico analyses, mRNA expression and related clinical data were obtained from the Molecular Taxonomy of Breast Cancer International Consortium (METABRIC) dataset [26, 27] and The Cancer Genome Atlas (TCGA) Pan-Cancer Atlas via cBioportal [28]. Breast cancer subtypes were classified according to the PAM50 signature [29]. Extreme quartiles were plotted in Kaplan-Meier curves to determine the OS. The mRNA expression data for cell models were obtained from the Cancer Cell Line Encyclopedia via DepMap portal [30]. For the metabolome analyses, the HMDB [22] identified and categorized the metabolites. Kyoto Encyclopedia of Genes and Genomes (KEGG) [31] and Small Molecule Pathway Data Base (SMPDB) [32] were used to classify the metabolites into their corresponding metabolic pathways. MetaboAnalyst web tool [33] was employed for identifying metabolite-metabolite interactions. Metabolite interacting genes were determined in MetaboAnalyst and Metabolic Atlas [34]. STRING web tool [35] was used for uncovering gene interactions. Functional analysis for biological processes was performed in Gene Set Enrichment Analysis (GSEA) [36].

### Statistical analysis

The results are presented as mean ± standard deviation (SD) or error of the mean (SEM) of at least three independent biological experiments (indicated as n). Statistical significance was determined using an unpaired two-tailed Student’s t-test or one-way ANOVA, followed by post-hoc analysis employing Tukey’s or Dunnet´s methods. Prism8 (GraphPad) or Microsoft Excel 2016 was used for statistical analyses. For Kaplan-Meier curve analysis the log-rank test was used and results with *p* > 0.05 were defined as significant. Untargeted metabolome data processing and statistics were performed on the MetaboAnalyst platform. Normalized metabolite peak intensity (relative to non-irradiated control) was transformed to ensure normal distribution and significantly altered metabolites were identified by t-test. Degree of correlation was determined by Pearson correlation using Python or Prism8.

## Results

### Ionizing radiation modulates the metabolome of TNBC cells

**Fig. 1:**
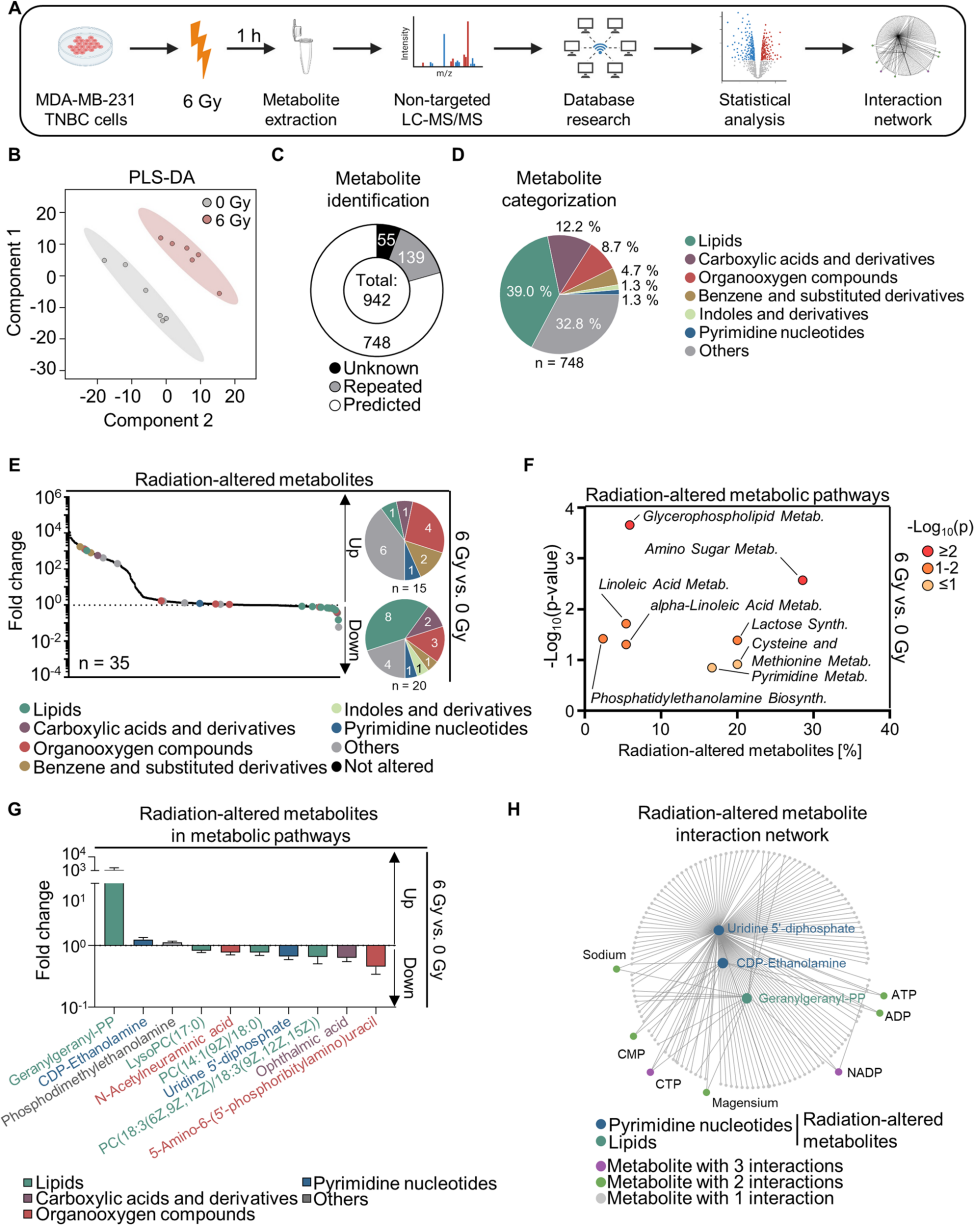
Metabolome characterization of TNBC cells after irradiation. **A** Workflow of untargeted metabolomics in irradiated cells. Image created with BioRender.com. **B** Partial Least-Squares Discriminant Analysis (PLS-DA) on metabolome dataset from MDA-MB-231 cells created using MetaboAnalyst.ca web tool. **C** LC-MS/MS analytical feature processing for metabolite prediction using Human Metabolome Data Base (HMDB) web tool. **D** Classification of metabolites into their respective categories using HMDB. **E** Identification of radiation-induced changes in metabolites categorized by color-coded groups. Data are presented as mean (*n* = 6) fold change relative to control, with significance determined by t-test (*p* < 0.05) in MetaboAnalyst.ca. Circles indicate the number of up- and downregulated metabolites in the respective category. **F** Impact of irradiation on metabolic pathways in TNBC cells. Corresponding p-values are aligned with the percentage of altered metabolites relative to all metabolites found in the indicated pathway. Data obtained from MetaboAnalyst.ca, Kyoto Encyclopedia of Genes and Genomes (KEGG), and Small Molecule Pathway Data Base (SMPDB) databases. **G** Relative abundance of metabolites altered in irradiated cells within the indicated metabolic pathways, presented as a mean ± SEM (*n* = 6) fold change relative to the control. **H** Metabolite-metabolite interaction network of metabolites altered in irradiated cells relative to controls, generated in MetaboAnalyst.ca

To explore radiation-induced metabolic changes putatively associated with resistance, we conducted untargeted metabolomics in irradiated MDA-MB-231 cells (Fig. 1A). Clearly distinguishable metabolome patterns were identified (Fig. 1B). Subsequently, we predicted and categorized 748 metabolites out of 942 measured features (Fig. 1C-D; Supplementary Table S2). In addition to the category others (32.8%), the top three categories were lipids (39%), carboxylic acids and derivatives (12.2%), and organooxygen compounds (8.7%) (Fig. 1D). We identified 35 metabolites with significant variations in abundance upon irradiation (Fig. 1E). The 15 upregulated metabolites were mostly belonging to the organooxygen compounds class and the 20 downregulated were predominantly categorized as lipids (Fig. 1E). KEGG and SMPDB-based pathway analyses revealed ten of the altered metabolites to be mainly part of either glycerophospholipid, amino sugar, lactose or cysteine and methionine metabolism (Fig. 1F). Figure 1G shows ten altered metabolites of the indicated pathways, which are either significantly up- (e.g. geranylgeranyl-PP) or downregulated (e.g. 5-amino-6-(5’-phosphoribitylamino)uracil) in irradiated relative to non-irradiated cells. Subsequent bioinformatic analyses for metabolite interaction networks unveiled that the above-mentioned metabolites connect to other metabolites identified in our untargeted metabolomics, such as nucleotides (ATP, ADP, CMP, CTP), NADP, magnesium, and sodium (Fig. 1H).

### Identification of potential therapeutic radiosensitizing targets based on radiation-induced metabolome alterations

**Fig. 2:**
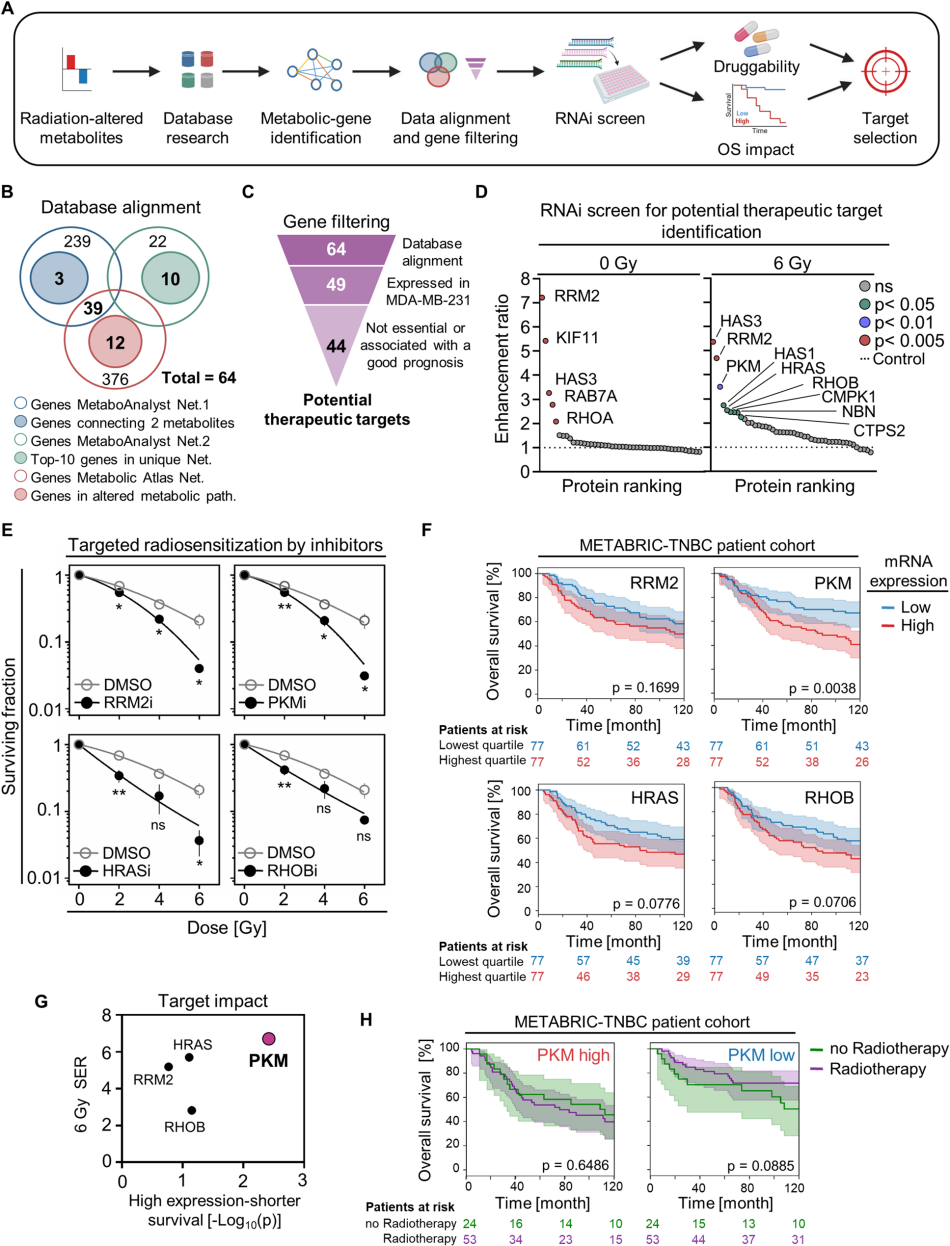
Identification of PKM as the most promising target for radiosensitization in TNBC cells.** A** Workflow illustrating the discovery of druggable candidates by database gene filtering on radiation-induced metabolic alterations. Image created with BioRender.com. **B** Venn diagram depicting the selection of metabolite-interacting genes through database alignment. **C** Gene filtering for the selection of potential therapeutic targets based on the indicated criteria. **D** Clonogenic basal survival (0 Gy) or clonogenic radiation survival (6 Gy) of MDA-MB-231 cells depleted of indicated candidates. Data are presented as enhancement ratios relative to non-specific siRNA controls, represented as mean (*n* = 4), and colored based on significance (p-value) calculated by one-way ANOVA. **E** Combination of the target-specific inhibitors (IC_50_) with irradiation (IR) in MDA-MB-231 cells (*n* = 3). Results show mean ± SEM. Statistical significance was determined by unpaired two-tailed Student’s t-test and indicated as * *p* < 0.05; ** *p* < 0.01, or ns = not significant. **F** Kaplan-Meier analyses based on the mRNA expression of the indicated candidate genes using the METABRIC-TNBC patient cohort. 10-year overall survival (OS) curves show the highest vs. the lowest mRNA expression quartiles, the confidence intervals, the log-rank test p-values and the patients at risk. **G** Comparative depiction of the radiosensitizing effect upon target inhibition in MDA-MB-231 cells and the reduced OS in TNBC patients with target high expression. The inhibitor effect is presented as the 6 Gy sensitizing enhancement ratio (SER), and the effect on patient OS is represented as the -Log_10_ (p-value) of the difference in patient OS between low and high expression. **H** Kaplan-Meier analyses using the METABRIC-TNBC patient cohort to assess the impact of radiotherapy (RT) on high and low PKM expression levels, with confidence intervals plotted, log-rank test p-values and patients at risk

To uncover potential therapeutic targets, we identified genes associated with metabolites altered by irradiation (Fig. 2A). Using open-access databases, a gene-metabolite interaction network was created, incorporating five of the ten altered metabolites (see Fig. 1G-H; Supplementary Fig. S1A). Gene alignment and filtering revealed a network of 44 genes based on the following criteria: (i) interactions with the five altered metabolites, (ii) expression in the studied TNBC cell model, (iii) categorization as non-essential gene, and (iv) no previously described impact as biomarker for a good prognosis (Fig. 2B-C; Supplementary Fig. S1B). Further, STRING-based computational analyses revealed several interactions among the majority of those genes (Supplementary Fig. S1C). Subsequent gene enrichment analysis unraveled the involvement of these genes in key biological processes, particularly related to carbohydrate, nucleotide, glycoprotein, and lipid metabolisms (Supplementary Fig. S1D).

To explore the role of these genes in the context of radiosensitization, we performed a RNAi screen to assess the colony-forming capacity of MDA-MB-231 cells (Fig. 2D). Gene depletion alone elicited cytotoxicity for RRM2, HAS3, RAB7A, and RHOA (enhancement ratios ranging between 2 and 7; KIF11 included as positive control) (Fig. 2D; Supplementary Fig. S2A; Supplementary Table S3). In combination with irradiation, depletion of HAS3, RRM2, PKM, HAS1, HRAS, RHOB, CMPK1, NBN, and CTPS2 mediated radiosensitization (enhancement ratios between 2 and 5) (Fig. 2D; Supplementary Fig. S2B; Supplementary Table S3). Gene Ontology analysis uncovered common functions for these potential targets primarily centered on carbohydrate and nucleotide metabolisms (Supplementary Fig. S2C).

### PKM shows highest radiosensitizing potential upon pharmacological inhibition in TNBC cells and METABRIC-TNBC patient cohort analyses

Pharmacological inhibition of the four druggable candidates (RRM2, PKM, HRAS, and RHOB) confirmed a cytotoxic and radiosensitizing potential in MDA-MB-231 cells (Fig. 2E; Supplementary Fig. S3A-B). However, METABRIC-TNBC patient cohort analysis revealed that solely high PKM expression was significantly associated with worse OS of TNBC patients (Fig. 2F). While across all breast cancer subtypes associations between high expression of RRM2 and HRAS with worse OS were also observed (Supplementary Fig. S4A). Furthermore, only PKM showed no correlation with known clinical confounders (Supplementary Fig. S4B-D). Consequently, by correlating the clinical relevance of the candidate genes with their radiosensitizing effects in TNBC cells, PKM emerged as the most promising therapeutic target (Fig. 2G). Moreover, TNBC patients with low PKM expression showed a trend towards benefitting from radiotherapy not quite reaching significance, while there is no difference in response to radiotherapy in TNBC patients with a high expression of PKM (Fig. 2H).

### Targeting PKM in combination with irradiation induces radiosensitization, and decreases cell viability and proliferation in TNBC cell models

**Fig. 3:**
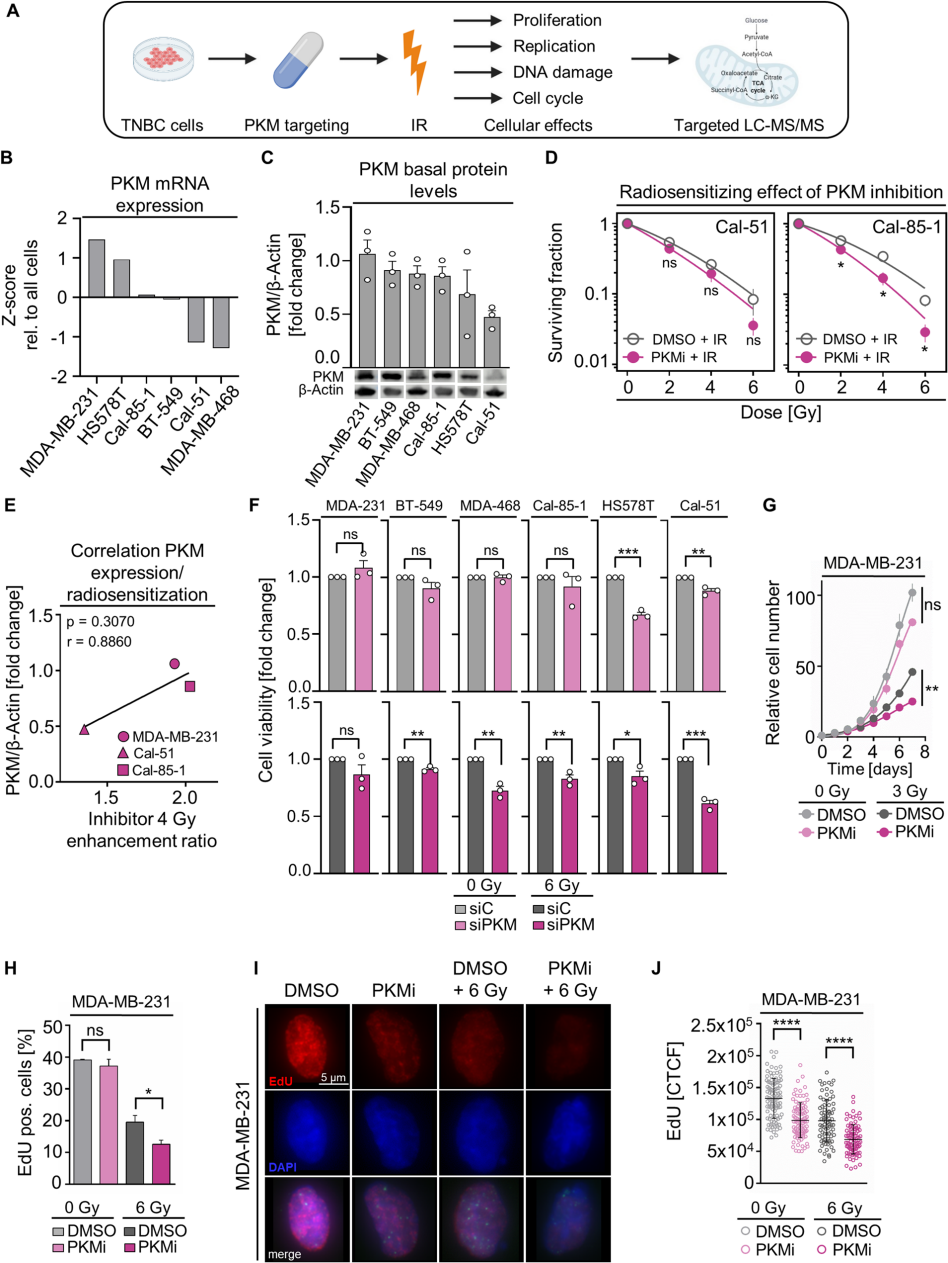
PKM inhibition compromises TNBC cell clonogenicity, viability and proliferation.** A** Workflow of functional analyses upon PKM targeting in combination with irradiation in TNBC cell models. Image created with BioRender.com. **B** Analysis of PKM mRNA expression in TNBC cells using the CCLE dataset. **C** Western blotting and densitometry from whole TNBC cell lysates to evaluate PKM levels in the indicated models (β-actin served as loading control). Protein levels were quantified using ImageJ software. **D** Clonogenic survival in irradiated Cal-51 and Cal-85-1 cells pretreated with IC_50_ of PKM inhibitor compound 3 K. **E** Correlation of PKM expression levels with enhancement ratio after 4 Gy irradiation in indicated cell models. **F** Cell viability measured by CellTiterGlo in non-irradiated (0 Gy) or irradiated (6 Gy) PKM-depleted TNBC cells. **G** Proliferation of MDA-MB-231 cells based on cell number counted from day one to seven after indicated treatments. **H** Quantification of EdU-positive cells relative to total cell count. At least 100 cells were counted in each biological replicate and condition. **I** Representative immunofluorescence images of EdU-positive cells with **J** associated quantification of the corrected total cell fluorescence (at least 100 cells per biological replicate). Mean values of three independent experiments ± SD or SEM are shown. Statistical significance was determined by unpaired two-tailed Student’s t-test and indicated as: * *p* < 0.05; ** *p* < 0.01; *** *p* < 0.001; **** *p* < 0.0001; or *ns* not significant

To better understand the role of PKM in the radiation response of TNBC cells, we performed functional analyses upon pharmacological and genetic inhibition in combination with irradiation in TNBC cell models with varying PKM expression (Fig. 3A-C). The radiosensitizing effect of PKM inhibition was confirmed in additional TNBC cell models (Fig. 3D; Supplementary Fig. S5A-B) and correlated with their PKM expression (Fig. 3E). Genetic PKM depletion alone led to a significant reduction in cell viability in two out of six TNBC cell models, whereas combined PKM depletion and irradiation resulted in diminished cell viability in five out of six analyzed TNBC cell models (Fig. 3F; Supplementary Fig. S5C). Consistently, while single PKM inhibition only marginally affected proliferation, PKM depletion plus irradiation elicited a significant antimitogenic effect relative to irradiation alone (Fig. 3G).

Given the reduced proliferation in TNBC cells, we next examined the impact of PKM inhibition on DNA replication. While the percentage of actively replicating EdU-positive cells remained unaffected after PKM inhibitor monotherapy, PKM inhibitor/irradiation induced a significant decline of EdU-positive cells (Fig. 3H). By observing significantly diminished values of the corrected total cell fluorescence of EdU (total number of replicating cells remained stable), we identified a lesser or slower incorporation of nucleotides under PKM inhibition, especially in irradiated cells (Fig. 3I-J).

### PKM inhibition modulates the radiation response in TNBC cells by regulating DNA damage repair, cell cycle progression, and DNA replication

**Fig. 4:**
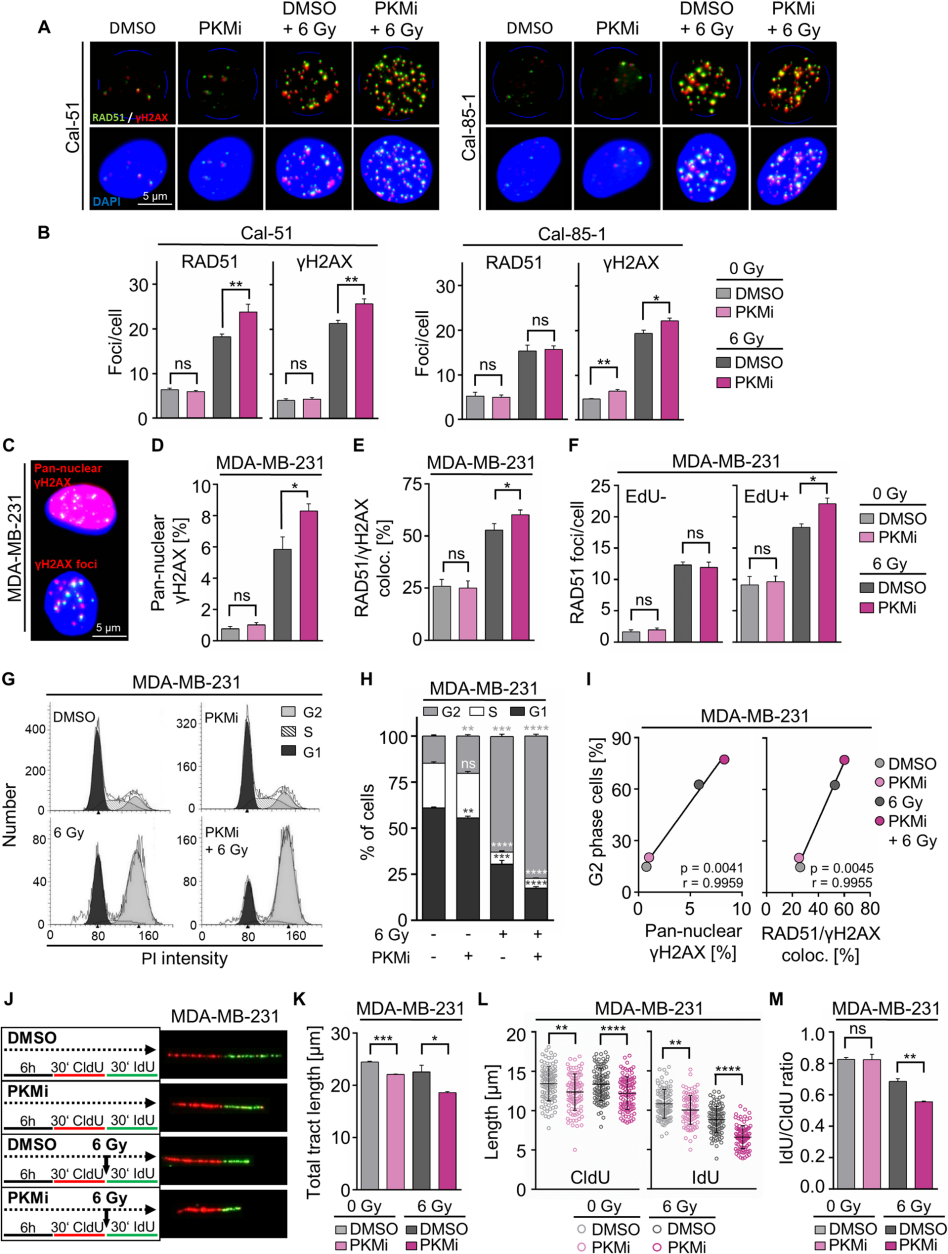
PKM inhibition modulates DNA damage repair, DNA replication, and induces cell cycle arrest in irradiated TNBC cells. **A** Representative immunofluorescence images displaying RAD51 (green) and γH2AX (red) co-stained foci upon indicated treatment. Nuclei stained with DAPI (40× magnification). **B** Separated quantification of RAD51 and γH2AX foci using the Aklides^®^ NUK system (40×magnification). At least 100 cells were analyzed in each biological replicate. **C** Representative immunofluorescence images of pan-nuclear γH2AX staining with **D** associated manual quantification from Aklides images. **E** Determination of RAD51/γH2AX colocalization using the Aklides^®^ NUK system after indicated treatment. **F** Quantification of RAD51 foci per EdU-negative or -positive cell. **G** Cell cycle profiles with corresponding **H** quantification of MDA-MB-231 cells measured by flow cytometry upon propidium iodide staining. **I** Correlation of G2 phase cells (FACS analysis) with pan-nuclear γH2AX signal or colocalization of RAD51/γH2AX foci with corresponding Pearson coefficient (r) and p-value. **J** Treatment scheme for DNA fiber assay with representative immunofluorescence images for each treatment. **K** Total tract length of DNA fiber measured with ImageJ. At least 100 fibers were measured per biological replicate. **L** Tract length of each individual label of the DNA fiber. **M** Ratio of IdU and CldU tract length. Mean values of three independent experiments ± SD or SEM are shown. Statistical significance was determined by unpaired two-tailed Student’s t-test and indicated as: * *p* < 0.05; ** *p* < 0.01; *** *p* < 0.001; **** *p* < 0.0001; or *ns* not significant

The fact that alterations in proliferation and DNA replication may be associated with replication stress and DNA damage prompted us to investigated these endpoints, shedding new light on the roles of PKM. While inhibitory PKM monotherapy and combined PKMi/irradiation failed to result in consistent modification of DNA damage markers (i.e. RAD51, γH2AX, and 53BP1) in all tested cell models (Supplementary Fig. S6A-E), γH2AX foci per cell were significantly elevated in Cal-51 and Cal-85-1 cells upon combined PKMi/irradiation compared to irradiation alone (Fig. 4A-B). Although the amount of total RAD51 and γH2AX was not increased in MDA-MB-231 cells after combined treatment (Supplementary Fig. S6B-C), a significant increase in the pan-nuclear γH2AX signal was observed in MDA-MB-231 and Cal-85-1 cells after PKM inhibition plus irradiation (Fig. 4C-D; Supplementary Fig. S6F-G); a finding similarly true for RAD51/γH2AX foci colocalization in MDA-MB-231 and Cal-85-1 cells (Fig. 4E; Supplementary Fig. S6H). Interestingly, the accumulation of DNA damage was replication-dependent indicative by significantly elevated RAD51 foci numbers in PKMi/irradiation exposed and EdU-positive MDA-MB-231 cells (Fig. 4F). In line, significant G2 phase accumulation occurred in MDA-MB231 and Cal-85-1 cells upon combined treatment (Fig. 4G-H; Supplementary Fig. S7A); however, elevated cyclin A, reduced cyclin D1, and increased inhibitory phosphorylation of CDK2 at T14 were not further enhanced by PKM inhibition under irradiated conditions (Supplementary Fig. S7B-C). Intriguingly, Pearson correlation analyses showed a significant association between the induced G2 arrest and the pan-nuclear γH2AX signal as well as the RAD51/γH2AX foci colocalization (Fig. 4I; Supplementary Fig. S7D-E).

We next examined DNA replication by means of the DNA fiber assay, which revealed a significant reduction in replication tract length upon PKM inhibition (Fig. 4J-K). Analysis of the CldU and IdU incorporation alone showed the same effect of tract length shortening after PKM inhibition. As irradiation was applied after the first labeling pulse, CldU tract length remained unchanged, indicating preserved fork stability. IdU tracts were substantially shortened by irradiation, with a further decrease by PKMi pre-treatment (Fig. 4L). Accordingly, the IdU/CldU ratio was unaffected by PKMi monotherapy, but significantly reduced in the combined treatment compared to irradiation alone (Fig. 4M). These observations suggest increased DNA replication stress when PKMi is co-applied to irradiation.

### PKM depletion modulates glycolysis and TCA cycle metabolite levels after irradiation in TNBC cells

**Fig. 5:**
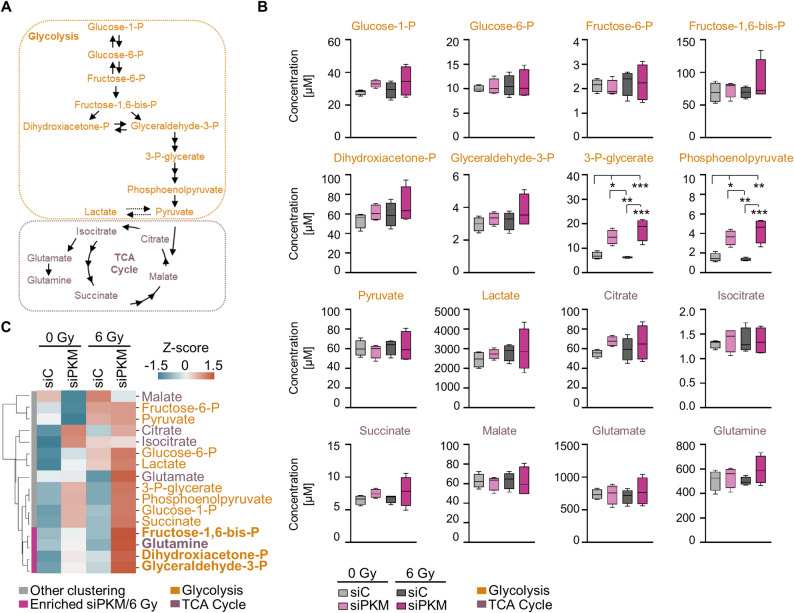
PKM depletion and irradiation modulate glycolysis and TCA cycle metabolite levels. **A** Mapping of studied metabolites from glycolysis and TCA cycle employing targeted metabolomics. **B** Depiction of metabolite levels upon indicated treatments in MDA-MB-231 cells. Metabolites were identified by targeted LC/MS-MS metabolomics. Data show metabolite concentrations (*n* = 4; one-way ANOVA; *p < 0.05; **p < 0.01; ***p < 0.005). **C** Heatmap showing the differential concentrations of metabolites in glycolysis and the TCA cycle following PKM depletion and irradiation. Data are presented as mean (*n* = 4) and ordered by ward clustering using Python

Given the impact of PKM depletion on multiple cellular functions and its metabolic role, we conducted targeted metabolomics to analyze alterations in relevant metabolites from glycolysis and TCA cycle (Fig. 5A). PKM depletion led to a significant accumulation of its substrate, phosphoenolpyruvate, along with a similar increase in the upstream metabolite, 3-P-glycerate (Fig. 5B). A non-significant but increasing trend was observed for the glycolysis-related metabolites dihydroxyacetone-P, fructose-1,6-bis-P and glyceraldehyde-3-P, as well as the TCA cycle metabolite glutamine. These metabolites formed an accumulated cluster upon PKM depletion and irradiation (Fig. 5B-C), while the final PKM product, pyruvate, or other metabolites from glycolysis and TCA cycle remained largely unaltered (Fig. 5B).

### High expression of PKM-associated gene clusters is linked to lower TNBC patient survival rates

**Fig. 6:**
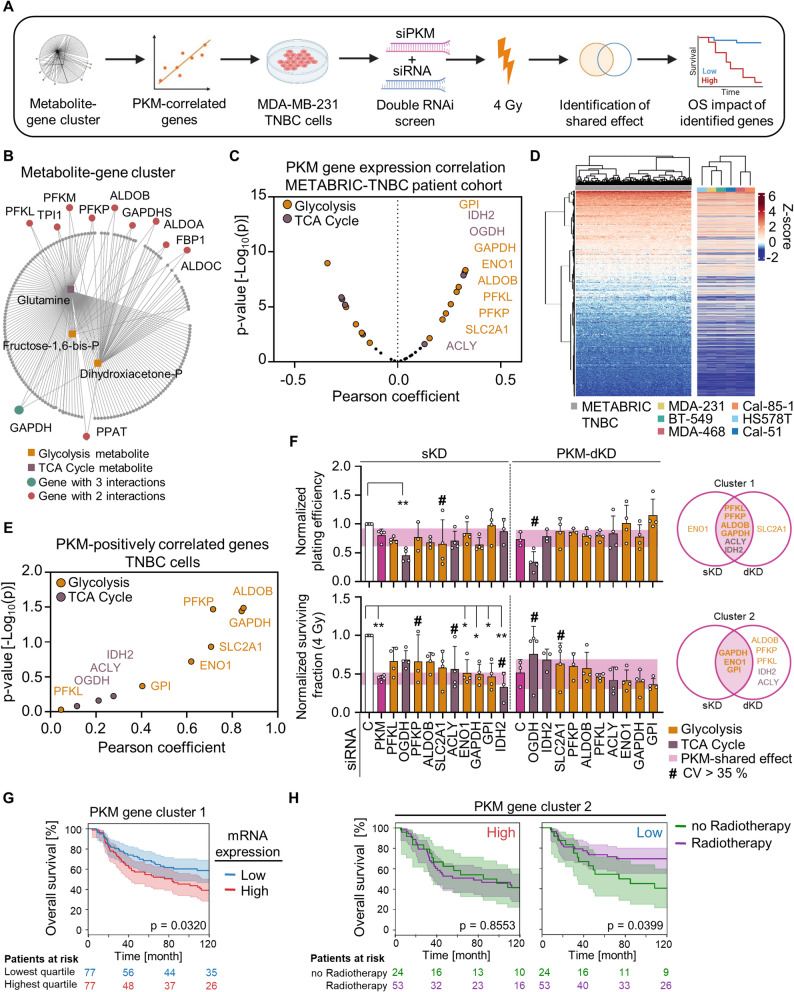
Glycolysis and TCA cycle enzymes share PKM effects on TNBC cell survival and radiation response.** A** Workflow of glycolysis and TCA cycle enzyme evaluation for a shared effect with PKM. Image created with BioRender.com. **B** Metabolite-gene interaction network of metabolites enriched upon PKM depletion and irradiation generated in MetaboAnalyst.ca. **C** Gene expression correlation between PKM and the selected enzymes in METABRIC-TNBC patient cohort. PKM significantly positively correlated genes are labelled. Pearson coefficients and p-values were calculated using Python. **D** Alignment of gene expression in METABRIC-TNBC patient cohort and the studied cell models from Cancer Cell Line Encyclopedia (CCLE). Heatmap was created with R, and data were hierarchically clustered (Ward.2) in rows based on patient gene expression, and in columns based on each heatmap samples. **E** Correlation analysis of gene expression between PKM and the labelled genes in ‘Fig. 6A’ in TNBC cell models. Data was obtained from CCLE, and Pearson coefficients and p-values were calculated using Prism. **F** Plating efficiencies and survival fractions of MDA-MB-231 cells upon single or PKM-double knockdown of the indicated enzymes. Normalized values (to control; *n* ≥ 3) are presented as mean ± SD and analyzed using one-way ANOVA and indicated as: * *p* < 0.05; ** *p* < 0.01. Single and double knockdown shared effects (within the PKM area) with a coefficient of variance (CV) lower than 35% are overlapped for 0 and 4 Gy X-ray irradiation. **G** Kaplan-Meier analysis based on the co-expression of cluster 1 from ‘Fig. 6F’ using the METABRIC-TNBC patient cohort. OS curves plot the highest vs. the lowest mRNA expression, confidence intervals, log-rank test p-values and patients at risk. **H** Kaplan-Meier analyses using the METABRIC-TNBC patient cohort to assess the impact of RT on high and low cluster 2 expression levels, confidence intervals, log-rank test p-values and patients at risk are plotted

Finally, we focused on the PKM-altered metabolome under irradiated conditions to evaluate the involvement of key glycolytic and TCA cycle enzymes in the context of PKM-induced radiosensitization (Fig. 6A). Metabolic genes altered after PKM inhibition and interacting with accumulated metabolites from glycolysis and the TCA cycle (Fig. 6B; Supplementary Fig. S8A) plus other glycolytic genes relevant for cancer metabolism (SCL2A1 and HK2), were investigated for co-expression with PKM in the METABRIC-TNBC patient cohort. We found 10 out of the 34 selected genes showing a significantly positive correlation (Fig. 6C; Supplementary Fig. S8B). Notably, we observed similar gene expression patterns between the METABRIC-TNBC patient cohort and TNBC cell models from the Cancer Cell Line Encyclopedia (CCLE) (Fig. 6D; Supplementary Fig. S8C). Gene expression correlation analysis in the cell models also revealed a positive correlation between PKM and the 10 genes identified in the METABRIC-TNBC patient cohort (Fig. 6E; Supplementary Fig. S9A).

Subsequently, we performed single and PKM-combined double knockdown of these enzymes and evaluated a potentially PKM-shared function in terms of clonogenic survival. We found a cluster of genes (termed cluster 1) composed of PFKL, PFKP, ALDOB, GAPDH, ACLY, and IDH2, with shared basal survival effects upon single knockdown, as well as PKM-combined double knockdown of these genes. Under irradiated conditions, an exclusive glycolytic cluster of genes (GAPDH, ENO1, and GPI; termed cluster 2) shared the PKM-associated radiosensitizing effect (Fig. 6F; Supplementary Fig. S9B-C). High co-expression of PKM and cluster 1 evidenced a significant difference in OS of TNBC patients in the METABRIC cohort (Fig. 6G). Furthermore, TNBC patients with low co-expression of PKM and cluster 2 receiving radiotherapy had significantly better survival compared to those with high co-expression (Fig. 6H). Interestingly, comparable survival associations were observed in Head and Neck Squamous Cell Carcinoma (HNSCC), supporting high PKM and PKM-associated cluster expression associated with poorer outcomes in another tumor types where radiotherapy is a primary treatment modality (Supplementary Fig. S10A-B).

## Discussion

Therapy resistance of cancer cells is caused by a variety of factors and processes, including the rewiring of the metabolism. Due to its proven potential to uncover therapeutic vulnerabilities, we investigated the metabolism in TNBC cells after irradiation. Here, we show that untargeted metabolomics is a promising tool for profiling the radiation-induced metabolic changes in TNBC cells. We constructed a metabolite-gene interaction network, identifying several druggable candidates with radiosensitizing potential, among which PKM emerged as the most promising target. Through genetic and pharmacological inhibition, we demonstrate how PKM influences TNBC cell survival, metabolism, particularly glycolysis and TCA cycle, modulates DNA replication and damage repair, and modifies cell cycling. Notably, a subset of glycolysis and TCA cycle-related genes mirrored the effects of PKM on cellular survival and radiation response, forming potential prognostic gene clusters linked to poorer overall survival and reduced response to radiotherapy in TNBC patients.

While the decoding of the cancer metabolome is still in its infancy, studies elucidating metabolic changes upon irradiation are even rarer. In our study, we uncovered alterations in phospholipids and pyrimidine nucleotides, primarily affecting glycerophospholipid and amino sugar metabolisms. These findings are consistent with previous studies where radiation elicited a dysregulation of metabolites from nucleotide synthesis, glycolysis, and TCA cycle across different breast cancer subtypes, including TNBC [37] and in patients [38]. In addition, increased amounts of antioxidants and phospholipids have been proposed as radioprotective agents, and reports of various cancer models that became resistant to repeated irradiation support this assumption [39]. Alterations in amino acid and fatty acid metabolisms, as well as changes in phospholipids, were similarly exhibited in other cancer entities such as glioma and head and neck upon radiotherapy [40, 41].

To follow through in terms of functionality, we asked whether we are able to connect the predicted altered metabolites from our untargeted metabolomics to potential therapeutic targets. Indeed, by means of an RNAi screen, we found nine genes with considerable potential as radiosensitizing targets. Six (i.e. HAS3, RRM2, PKM, HRAS, RHOB, NBN) of these nine genes were previously reported to have a function in the cellular radiation response of numerous cancer cell models [42–48]. The remaining three candidates (i.e. HAS1, CMPK1, CTPS2) presented novel in the context of modifying the cellular response to ionizing radiation. Among them, PKM showed the highest radiosensitizing potential based on our TNBC cell line analysis and retrospective analysis of the METABRIC-TNBC patient cohort.

Even though the isoform PKM2 is widely overexpressed across many cancer entities, making it a candidate prognostic marker in clinical trials (NCT03539731 [49], NCT01130584 [50]) and a viable target for small molecule inhibitors with radiosensitizing potential [51–53], recent findings have highlighted a significant role for the isoform PKM1 in cancer metabolism and resistance [14]. Thus, we did not distinguish between the isoforms and further investigated the impact of PKM inhibition by examining its effects on glycolysis and TCA cycle. Targeted metabolomics revealed an increase in PKM metabolic substrates: phosphoenolpyruvate and 3-P-glycerate upon its depletion. Additionally, we observed an enriched metabolite cluster (dihydroxyacetone-P, fructose-1,6-bis-P, glutamine, glyceraldehyde-3-P) after irradiation and PKM depletion. Previous studies have reported increased levels of glycolytic intermediates and TCA cycle metabolites as part of a metabolic adaptation in response to irradiation [54] and PKM2 depletion [53, 55], as well as changes in PKM2 expression upon irradiation [54].

The accumulation of glycolytic and TCA cycle intermediates has been described to directly interfere with DNA replication [16] and modulate DDR by altering nucleotide pool availability, redox balance, and the activity of DNA repair proteins [53, 56, 57]. In line, the accumulation of upstream glycolytic intermediates upon PKM depletion could modify enzymatic activity or impair the *de novo* synthesis of nucleotide precursors required for fork progression and effective DDR. Supporting this notion, previous reports have linked altered PKM metabolic signaling to the regulation of DNA replication dynamics in different model systems [58].

Indeed, in our cell models, PKM inhibition combined with irradiation resulted in increased pan-nuclear γH2AX and global deceleration of DNA replication, both hallmarks of replication stress [59, 60], as well as accumulation of cells in G2 phase. While PKM2 is a known substrate of ataxia-telangiectasia mutated (ATM) and promotes HR through phosphorylation of the C-terminal binding protein (CtBP)-interacting protein *(*CtIP) [18], our data suggest that this pathway is insufficient to resolve the damage under PKM inhibition. Specifically, the observed increase in RAD51/γH2AX colocalization likely reflects persistent recruitment of HR machinery proteins to stalled or collapsed replication forks rather than efficient repair completion, potentially due to a compromised metabolic environment. Collectively, these data support a model in which the metabolic perturbations induced by PKM inhibition could be exacerbating replication-associated DNA damage following irradiation, resulting in prolonged G2 arrest and the accumulation of unresolved genomic lesions.

Further, we identified a group of genes associated with the PKM-induced metabolome changes, correlated to PKM expression, and linked to PKM effects on cell survival and radiation response (i.e. ENO1, GAPDH, GPI; “cluster 2”). These glycolytic genes have been previously shown involvement in metastasis, adaptation to hypoxia, tumor aggressiveness, and cellular stress response [61–63]. Thus, they all contribute to reprogramming cancer metabolism, are associated with a bad prognosis, and may therefore contribute to therapy resistance. This is consistent with our observation that patients with a high expression of cluster 2 show a worse response to radiotherapy compared to those with low expression. Over the last years, few studies designed glycolysis-related gene signatures to predict recurrence [64], response to immunotherapy [65], and survival of breast cancer patients [66, 67]. In addition to other studies linking glycolysis-related gene signatures to therapeutic response in TNBC [68], we demonstrate for the first time that a glycolysis-related gene cluster holds prognostic value for radiotherapy outcomes. Remarkably, this prognostic value extends beyond TNBC to include patients with HNSCC, suggesting that this metabolic signature could serve as a potential biomarker for various tumor types where radiotherapy is a primary treatment modality. However, given the various limitations of retrospective analyses, including the selection criteria for radiotherapy, further validation in preclinical studies, and preferably in prospective studies, is urgently needed.

## Conclusion

Our study demonstrates that untargeted metabolomics is a promising tool to identify novel therapeutic targets. By delving into the metabolomic changes induced in irradiated TNBC cells, we uncovered potential clinically relevant targets whose inhibition could improve the response to radiotherapy in TNBC patients. We show that PKM and its associated genes are potential therapeutic vulnerabilities. Future work is warranted to clarify the therapeutic relevance of PKM and its related enzymes. Our research clearly supports the utility of metabolomics to improve cancer treatment.

## Supplementary Information

Supplementary Material 1.

Supplementary Material 2.

Supplementary Material 3.

Supplementary Material 4.

Supplementary Material 5.

## Data Availability

The datasets supporting the conclusions of this article are included within the article and its additional files. All data and materials will be made available to the scientific community upon request.

## Abbreviations

DDR: DNA damage response
HR: Homologous recombination
PKM: Pyruvate kinase M
TCA: Tricarboxylic acid
TNBC: Triple Negative Breast Cancer
