# Supplementary material for: Precise metabolomics identifies glycolysis-related pyruvate kinase M activity as regulator of the S-phase-specific radiation response in triple-negative breast cancer cells
Source: Cell Commun Signal. 2026 Mar 12;24:216. doi: 10.1186/s12964-026-02803-5 (PMC13064355; doi:10.1186/s12964-026-02803-5)
Supplement: Supplementary file 4 — Supplementary Material 4. [file 12964_2026_2803_MOESM4_ESM.pdf]

Fig. 3I

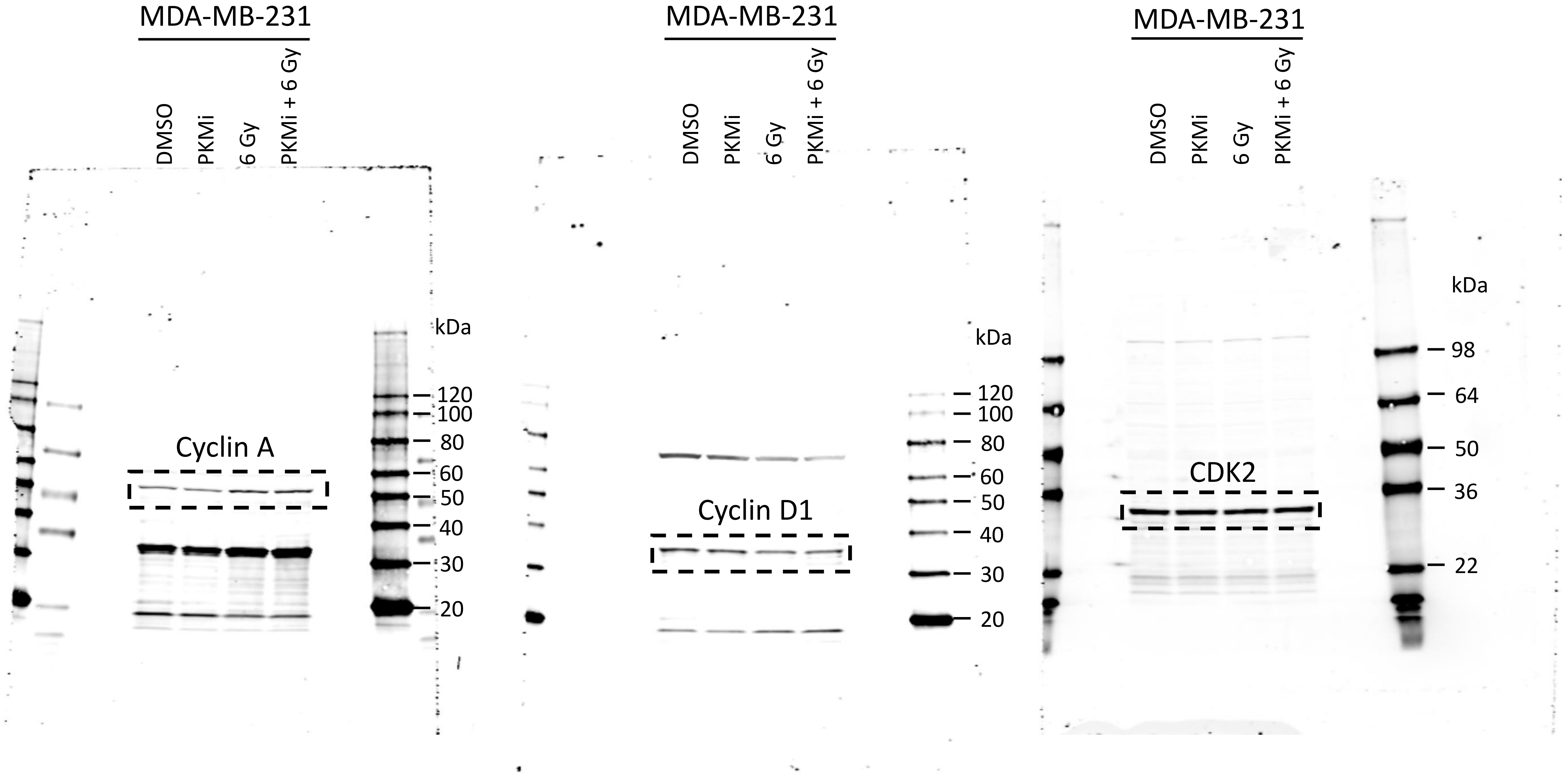
MDA-MB-231
DMSO
PKMi
6 Gy
PKMi + 6 Gy
kDa
120
100
80
60
50
40
30
20
Cyclin A
MDA-MB-231
DMSO
PKMi
6 Gy
PKMi + 6 Gy
kDa
120
100
80
60
50
40
30
20
Cyclin D1
MDA-MB-231
DMSO
PKMi
6 Gy
PKMi + 6 Gy
kDa
98
64
50
36
22
CDK2

Fig. 3I

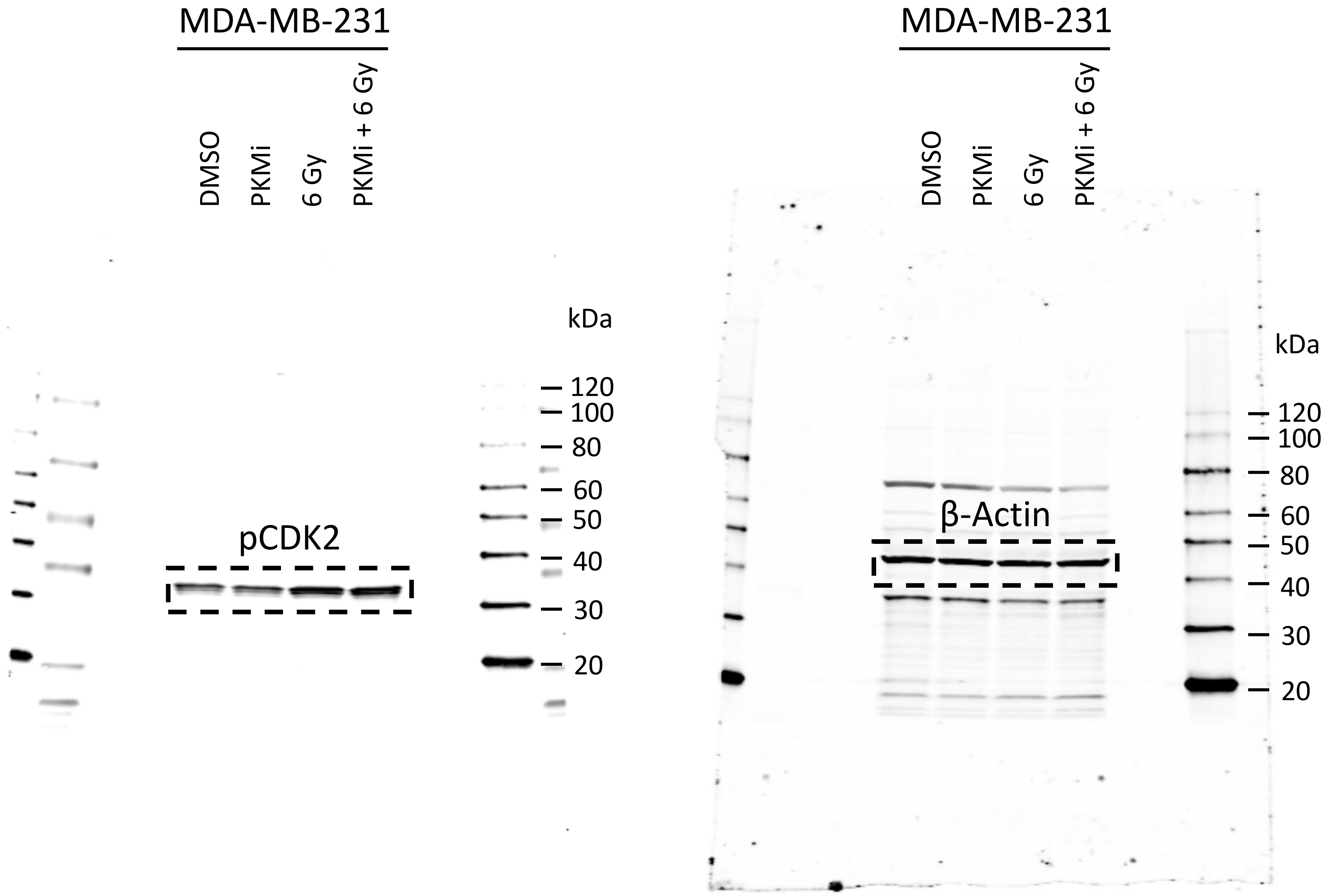
MDA-MB-231
DMSO
PKMi
6 Gy
PKMi + 6 Gy
pCDK2
kDa
120
100
80
60
50
40
30
20
MDA-MB-231
DMSO
PKMi
6 Gy
PKMi + 6 Gy
β-Actin
kDa
120
100
80
60
50
40
30
20

Fig. S5B

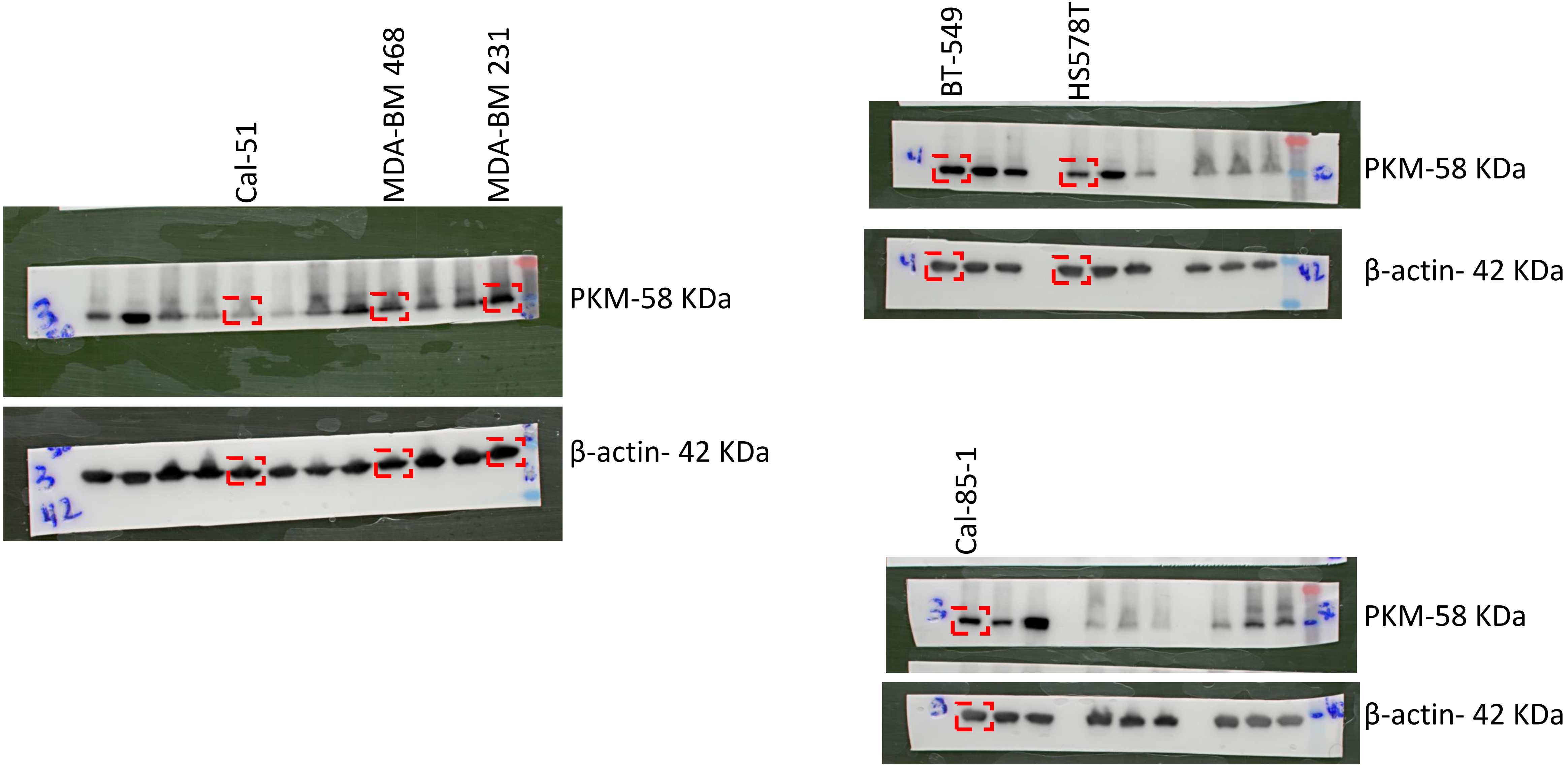
Cal-51
MDA-BM 468
MDA-BM 231
PKM-58 KDa
β-actin- 42 KDa
BT-549
HS578T
PKM-58 KDa
β-actin- 42 KDa
Cal-85-1
PKM-58 KDa
β-actin- 42 KDa

Fig. S5G

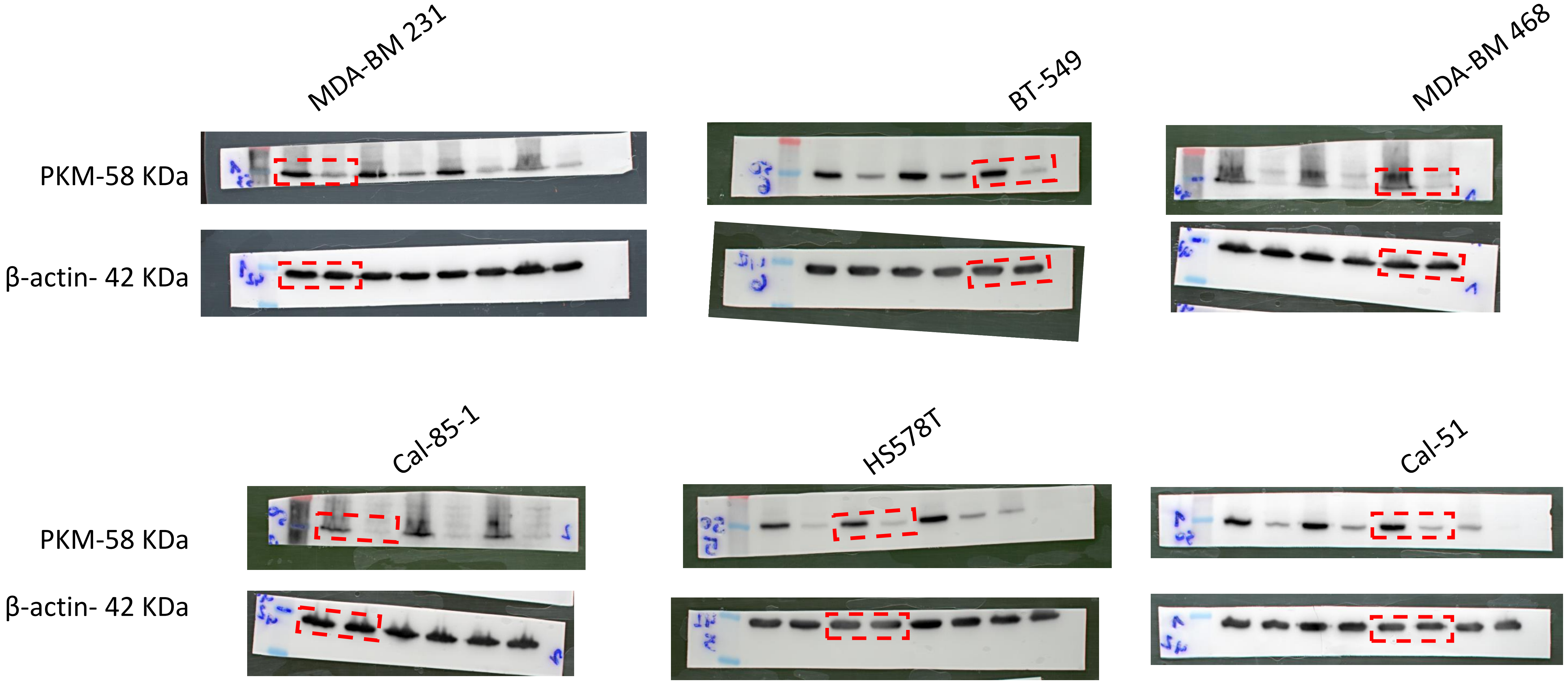
MDA-BM 231
BT-549
MDA-BM 468
PKM-58 KDa
β-actin- 42 KDa
Cal-85-1
HS578T
Cal-51
PKM-58 KDa
β-actin- 42 KDa

Fig. S6

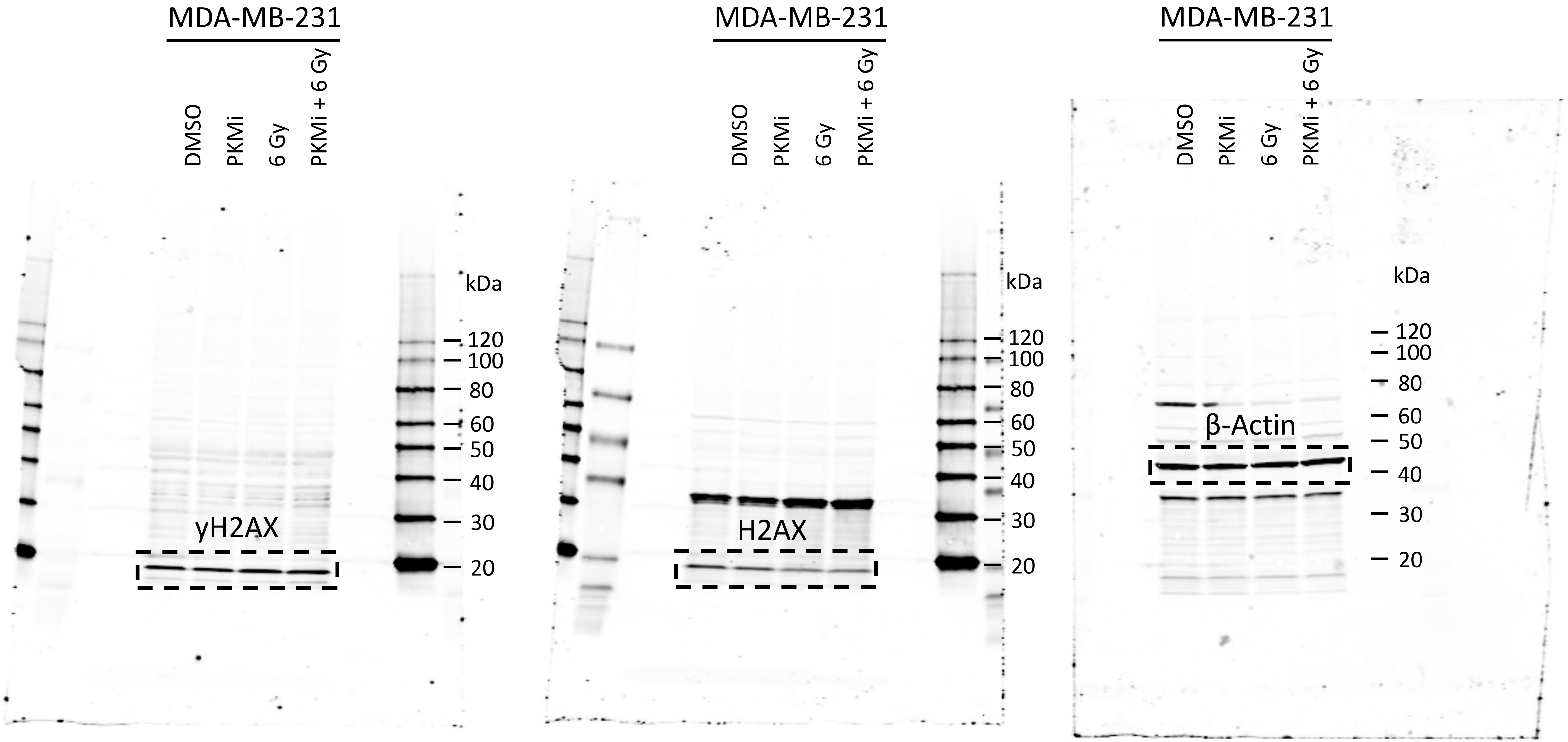
MDA-MB-231
DMSO
PKMi
6 Gy
PKMi + 6 Gy
kDa
120
100
80
60
50
40
30
20
yH2AX
MDA-MB-231
DMSO
PKMi
6 Gy
PKMi + 6 Gy
kDa
120
100
80
60
50
40
30
20
H2AX
MDA-MB-231
DMSO
PKMi
6 Gy
PKMi + 6 Gy
kDa
120
100
80
60
50
40
30
20
β-Actin
