# Supplementary material for: Precise metabolomics identifies glycolysis-related pyruvate kinase M activity as regulator of the S-phase-specific radiation response in triple-negative breast cancer cells
Source: Cell Commun Signal. 2026 Mar 12;24:216. doi: 10.1186/s12964-026-02803-5 (PMC13064355; doi:10.1186/s12964-026-02803-5)
Supplement: Supplementary file 5 — Supplementary Material 5. [file 12964_2026_2803_MOESM5_ESM.docx]

**Supplemental Information**

**Precise metabolomics identifies glycolysis-related pyruvate kinase M activity as regulator of the S-phase-specific radiation response in triple-negative breast cancer cells**

Rocío Matesanz-Sánchez, Sandra Classen, Kanstantsin Siniuk, Mirko Peitzsch, Tiago Alves, Helmut Pospiech, Kerstin Borgmann, and Nils Cordes

The file includes: Supplementary Figures and the corresponding figure legends (Fig. S1 – S10)

**Supplementary Figures**

**
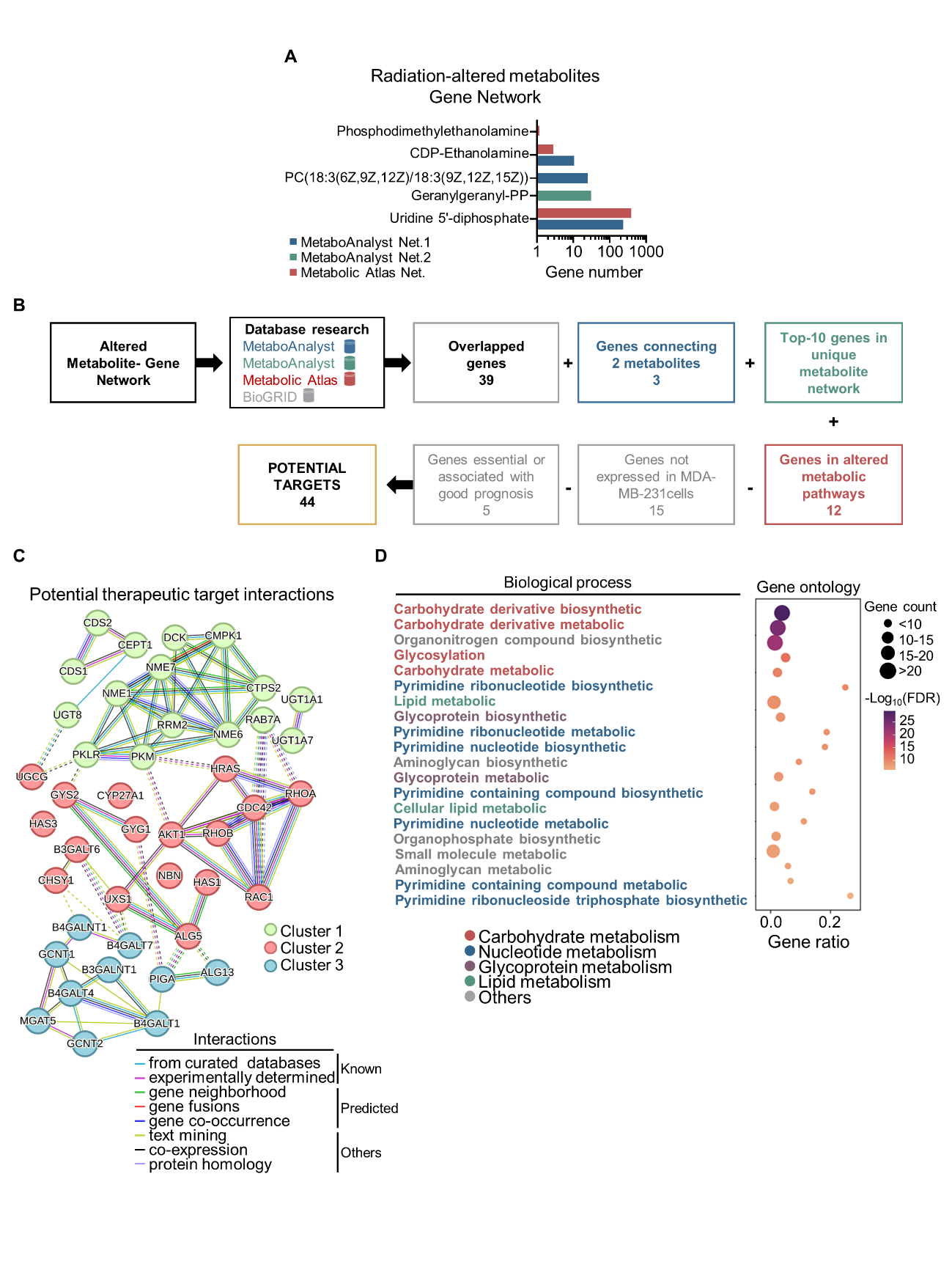
**

**Supplementary Fig. S1. Gene filtering for the selection of potential therapeutic targets based on radiation-induced metabolic alterations.** **(A)** Gene networks created with metabolites modified upon exposure to X-rays in MetaboAnalyst.ca and Metabolic Atlas. **(B)** Diagram illustrating the identification of potential targets through database filtering of genes selected based on radiation-induced metabolic alterations. **(C)** Potential candidate gene interaction network created by known, predicted and additional interactions as well as K-means clustering using STRING web tool. **(D)** Gene ontology analysis for the discovery of biological processes associated with potential gene candidates using gsea-msigdb.org.


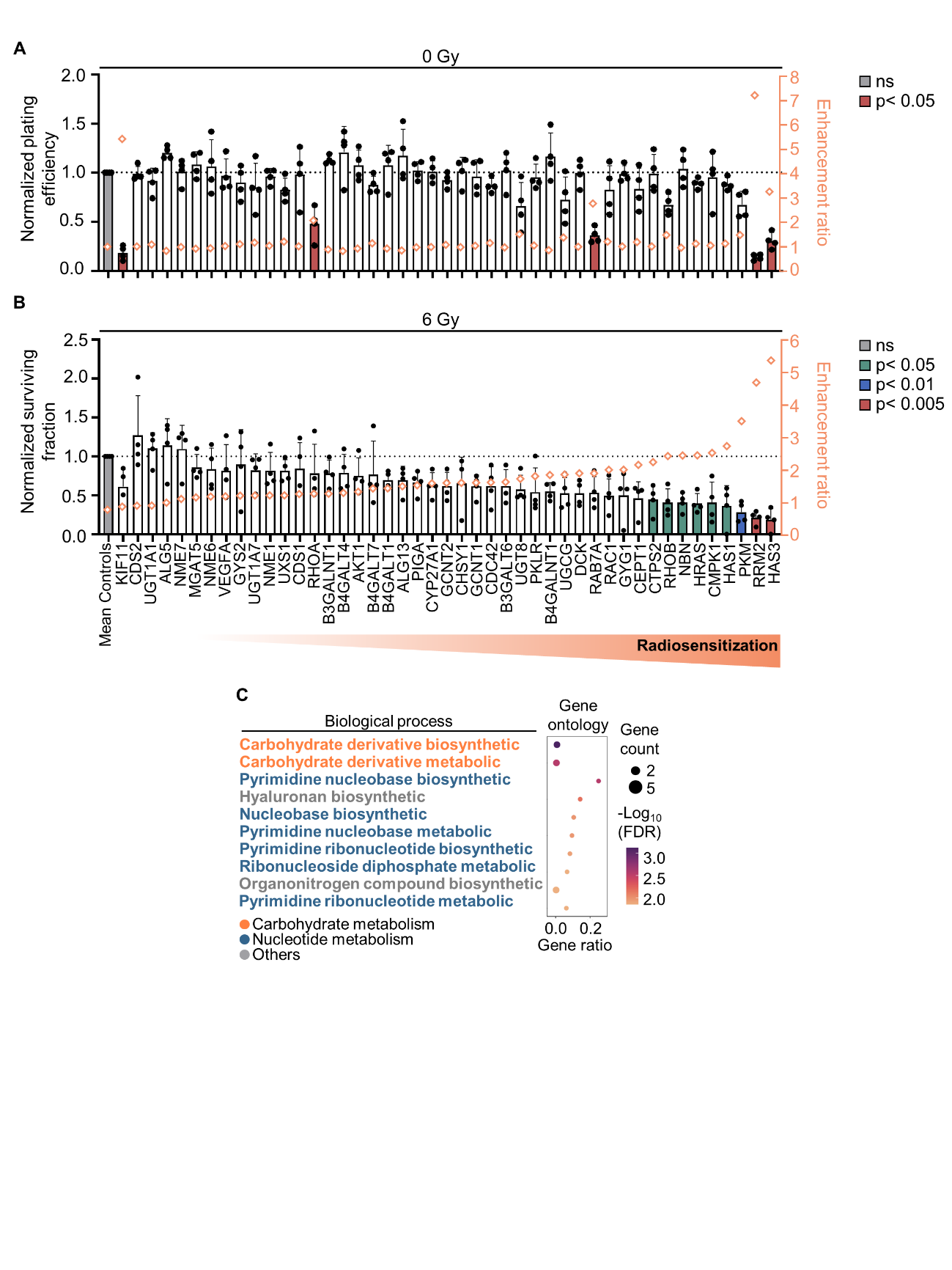


**Supplementary Fig. S2. RNAi screening for metabolism associated radiosensitizing targets in TNBC cells.** Clonogenic basal (plating efficiency) **(A)** and radiation survival **(B)** upon siRNA-mediated knockdown of indicated genes. Data are shown as mean ± SD (n = 4) of normalized values relative to controls (non-specific siRNA) and analyzed using one-way ANOVA. **(C)** Gene ontology analysis for biological process involvement of candidate genes identified in ‘**B’** using gsea-msigdb.org.


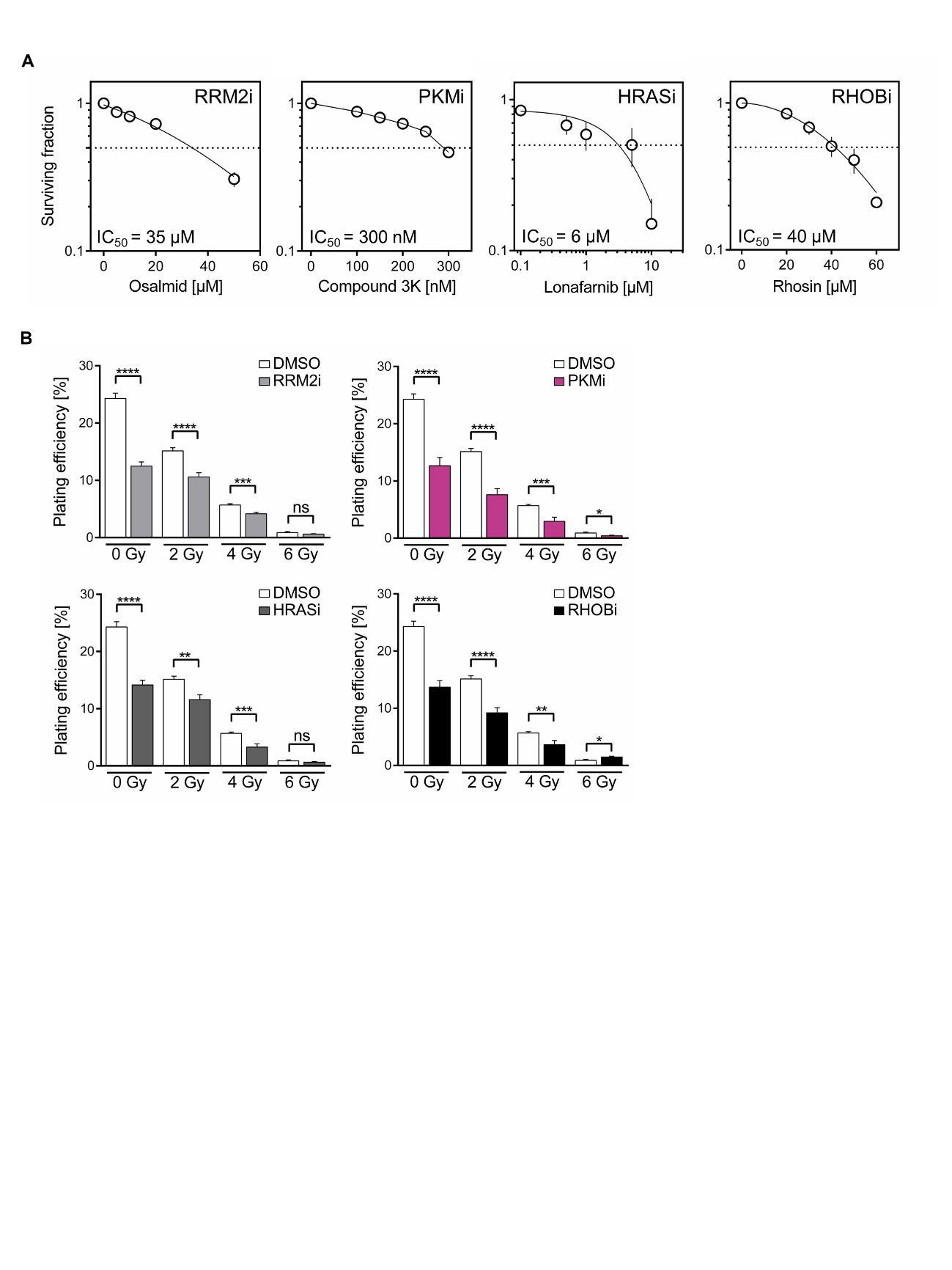


**Supplementary Fig. S3. PKM inhibition by compound 3K shows the promising radiosensitizing effect in TNBC cells**. **(A)** Titration of target-specific inhibitors in MDA-MB-231 cells to identify the corresponding IC_50_ using colony formation assay. **(B)** Plating efficiencies of MDA-MB-231 in response to irradiation and combined treatment with IC_50_ of target-specific drugs. Mean values of three independent experiments ± SEM are shown. Statistical significance was determined by unpaired two-tailed Student’s t-test and indicated as: * p < 0.05; ** p < 0.01; *** p < 0.001; **** p < 0.0001; or ns, not significant.


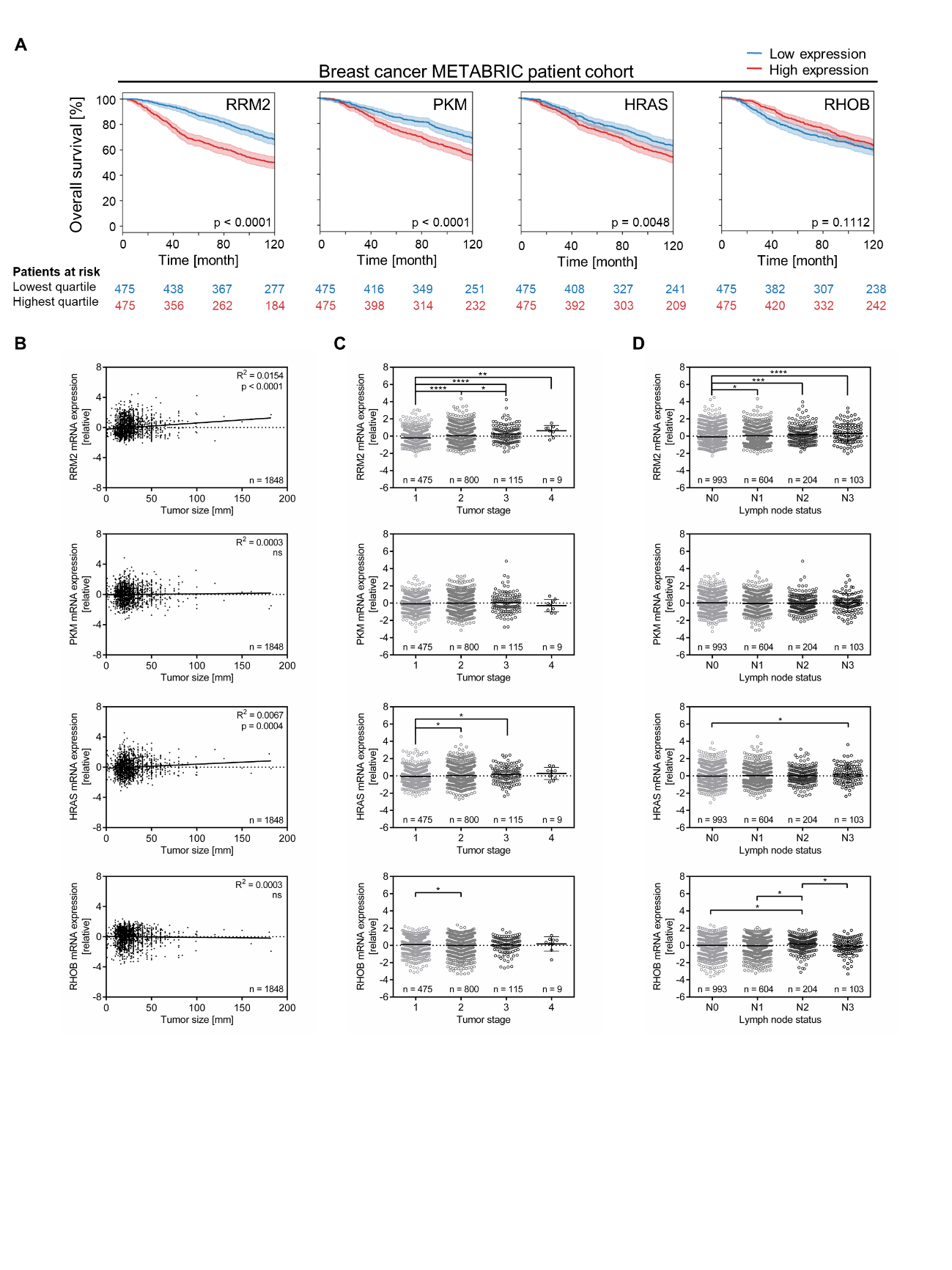


**Supplementary Fig. S4. METABRIC patient cohort analyses for target selection. (A)** Kaplan-Meier analyses of the highest and lowest mRNA expressing quartiles in relation to the 10-year OS of the METABRIC patient cohort, independent of the breast cancer subtype. Confidence intervals and patients at risk are indicated as well as significance was tested via log-rank test. **(B)** Linear regression analyses of tumor size relative to mRNA expression levels of identified target proteins in the METABRIC patient cohort. **(C)** mRNA expression levels of identified target proteins across different tumor stages. **(D)** mRNA expression levels of identified proteins dependent on lymph node status (N0: no positive lymph nodes; N1: 1-3 positive lymph nodes; N2: 4-9 positive lymph nodes; N3: >9 positive lymph nodes). Data in ‘**C’**, and ‘**D**’ are shown as mean ± SD and were analyzed using unpaired two-tailed Student’s t-test.


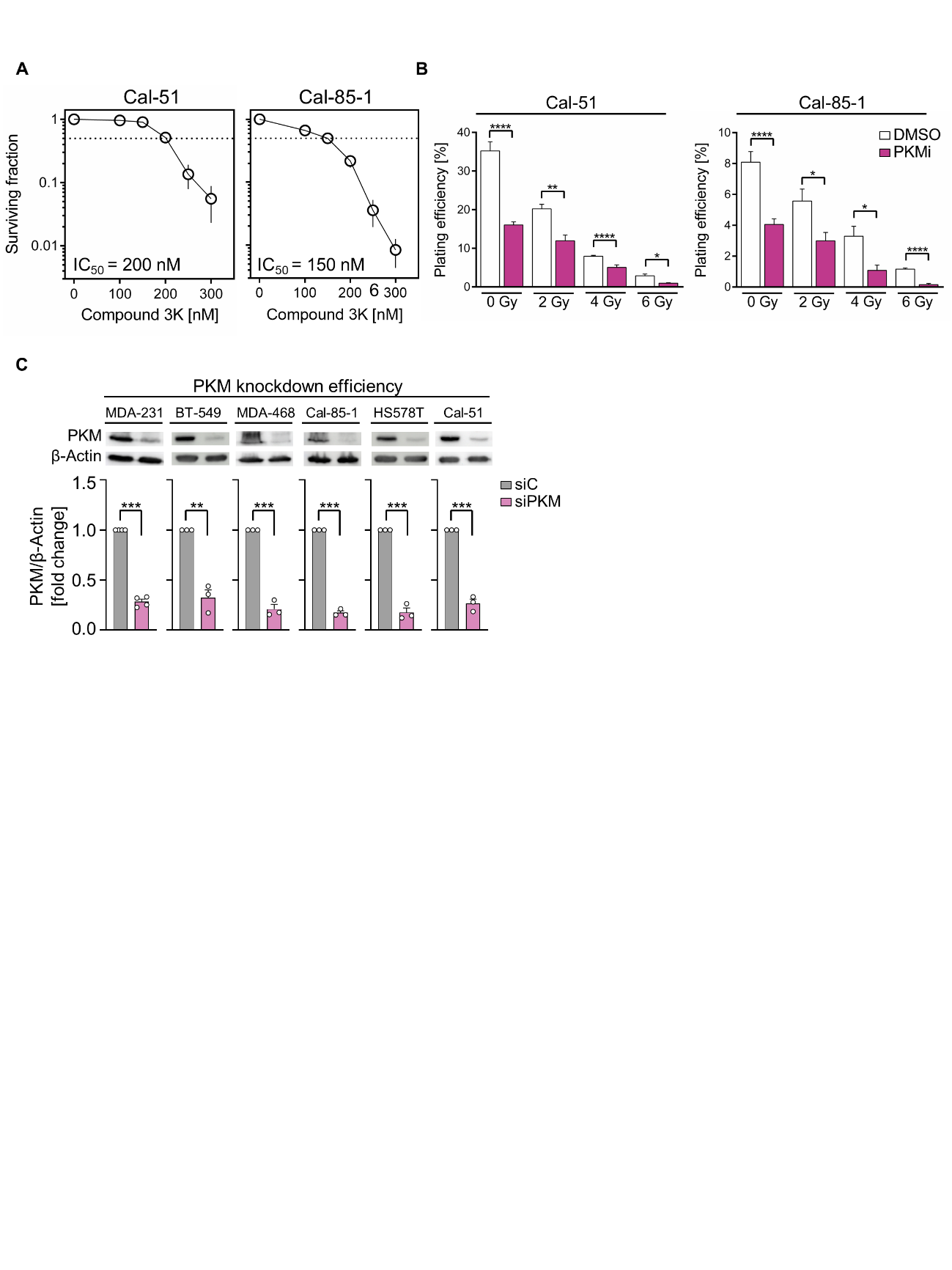


**Supplementary Fig. S5. Effects of PKM depletion across multiple TNBC cell lines.** **(A)** Titration of the PKM inhibitor compound 3K in different TNBC cell lines using colony formation assay. **(B)** Plating efficiencies of indicated cells in response to irradiation alone and combined with IC_50_ of PKM inhibitor compound 3K. **(C)** Western blotting and densitometry from whole TNBC cell lysates to evaluate PKM knockdown efficacy in the indicated models (β-actin served as loading control). Protein levels were quantified using ImageJ software. Mean values of three independent experiments. Data are presented as mean ± SD or mean ± SEM in ‘**B’**. Statistical significance was determined by unpaired two-tailed Student’s t-test and indicated as: * p < 0.05; ** p < 0.01; *** p < 0.001; **** p < 0.0001; or ns, not significant.


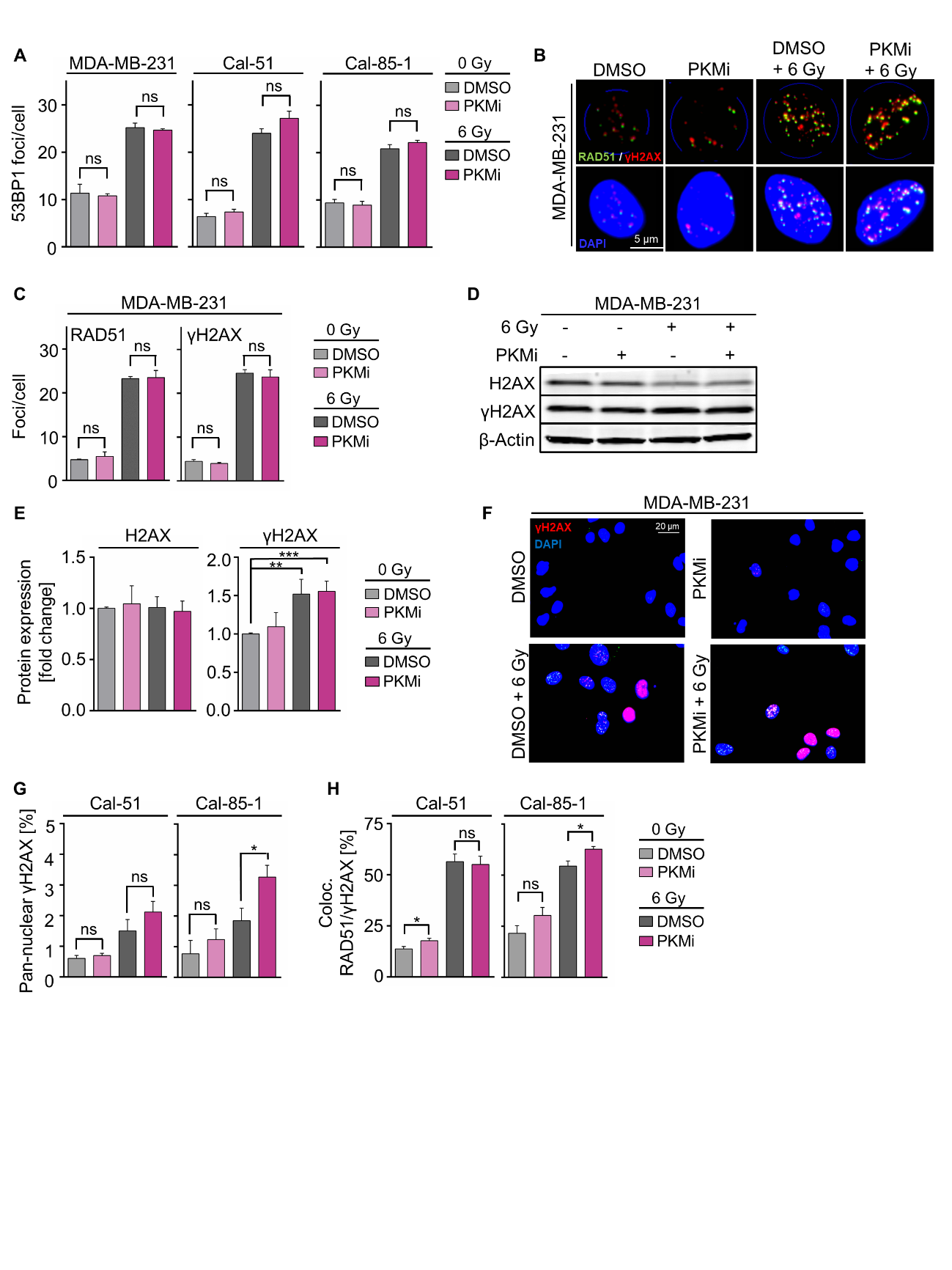


**Supplementary Fig. S6.** **Effects of PKM inhibition on DNA damage. (A)** Quantification of 53BP1 foci using the Aklides® NUK system (40× magnification). At least 100 cells were analyzed in each biological replicate. **(B)** Representative immunofluorescence images displaying RAD51 (green) and yH2AX (red) foci upon indicated treatment. Nuclei stained with DAPI. Pictures were taken on a 40× magnification. **(C)** Quantification of RAD51 and γH2AX foci using the Aklides® NUK system (40× magnification). At least 100 cells were analyzed in each biological replicate. **(D)** Representative western blot images of whole cell lysates of H2AX and γH2AX after indicated treatment in MDA-MB-231 cells (β-actin served as loading control). **(E)** Quantification of H2AX and γH2AX by densitometry, normalized to β-actin. Fold change relative to DMSO control. **(F)** Representative immunofluorescence images displaying pan-nuclear γH2AX signals (red) after indicated treatments in MDA-MB-231 cells. Nuclei stained with DAPI. Pictures were taken at 40× magnification. **(G)** Quantification of pan-nuclear γH2AX signal counted manually from the obtained Aklides® pictures of Cal-51 and Cal-85-1 cells. **(H)** Determination of RAD51/γH2AX colocalization using the Aklides® NUK system after indicated treatment. Mean values of three independent experiments ± SEM are shown. Statistical significance was determined by unpaired two-tailed Student’s t-test and indicated as: * p < 0.05; ** p < 0.01; *** p < 0.001; or ns, not significant.

**
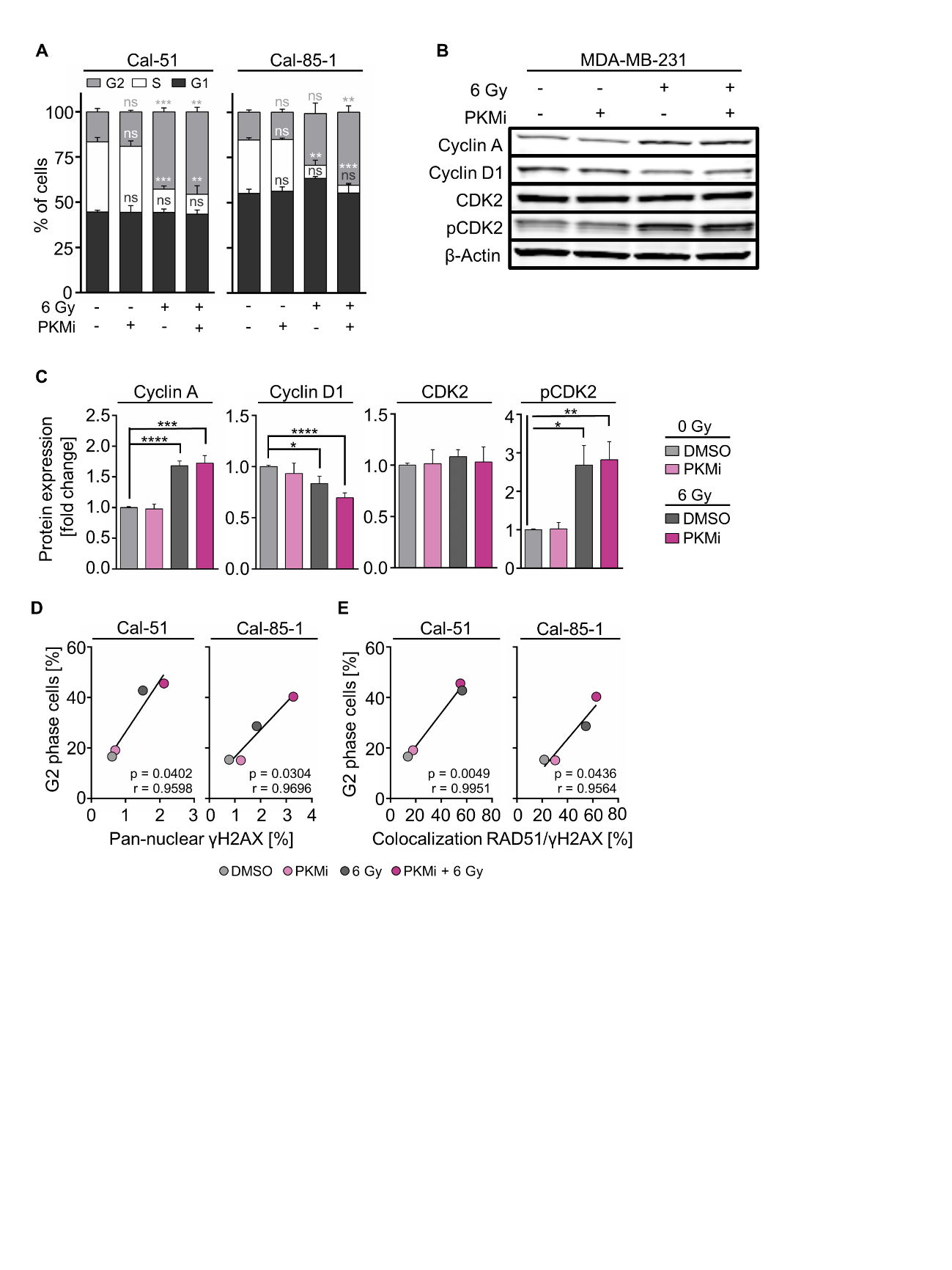
**

**Supplementary Fig. S7.** **Effects of PKM inhibition on cell cycle. (A)** Quantification of cell cycle profiles of Cal-51 and Cal-85-1 cells measured by flow cytometry upon propidium iodide staining. **(B)** Representative western blot images of whole cell lysates of cell cycle proteins after indicated treatment in MDA-MB-231 cells (β-actin served as loading control). **(C)** Quantification of cell cycle proteins by densitometry, normalized to β-actin. Fold change relative to DMSO control. **(D)** Correlation of G2 phase cells with pan-nuclear γH2AX with corresponding Pearson correlation coefficient (r) and p-value. **(E)** Correlation of G2 phase cells with colocalization of RAD51/γH2AX foci with corresponding Pearson correlation coefficient (r) and p-value for Cal-51 and Cal-85-1 cells. Mean values of three independent experiments ± SEM are shown. Statistical significance was determined by unpaired two-tailed Student’s t-test and indicated as: * p < 0.05; ** p < 0.01; *** p < 0.001; **** p < 0.0001 or ns, not significant.

**
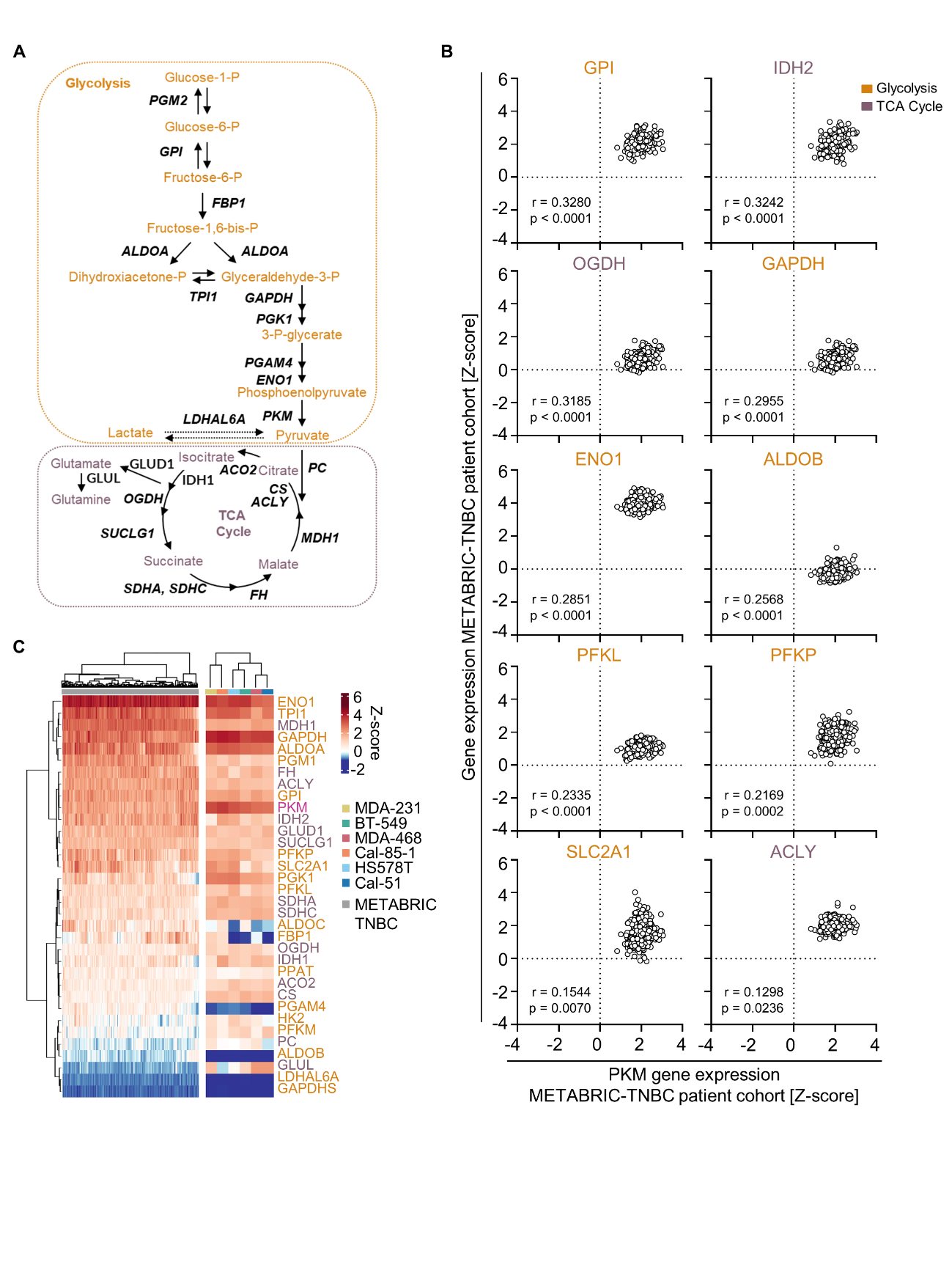
**

**Supplementary Fig. S8. Glycolysis and TCA cycle gene expression in TNBC. (A)** Mapping of glycolysis and TCA cycle enzymes. **(B)** Gene expression correlation analysis of PKM and genes identified in ‘**Fig. 6C**’ in METABRIC-TNBC patient cohort. Data obtained from Cbioportal.org are presented as Z-scores. Correlation was determined using the Pearson correlation coefficient (r) and the corresponding p-value, as indicated. **(C)** Alignment of gene expression in METABRIC-TNBC patient cohort and TNBC cell models from CCLE. Specific genes are indicated. Heatmap was created with R, and data were hierarchically clustered (Ward.2) in rows based on METABRIC data and in columns based on samples.

**
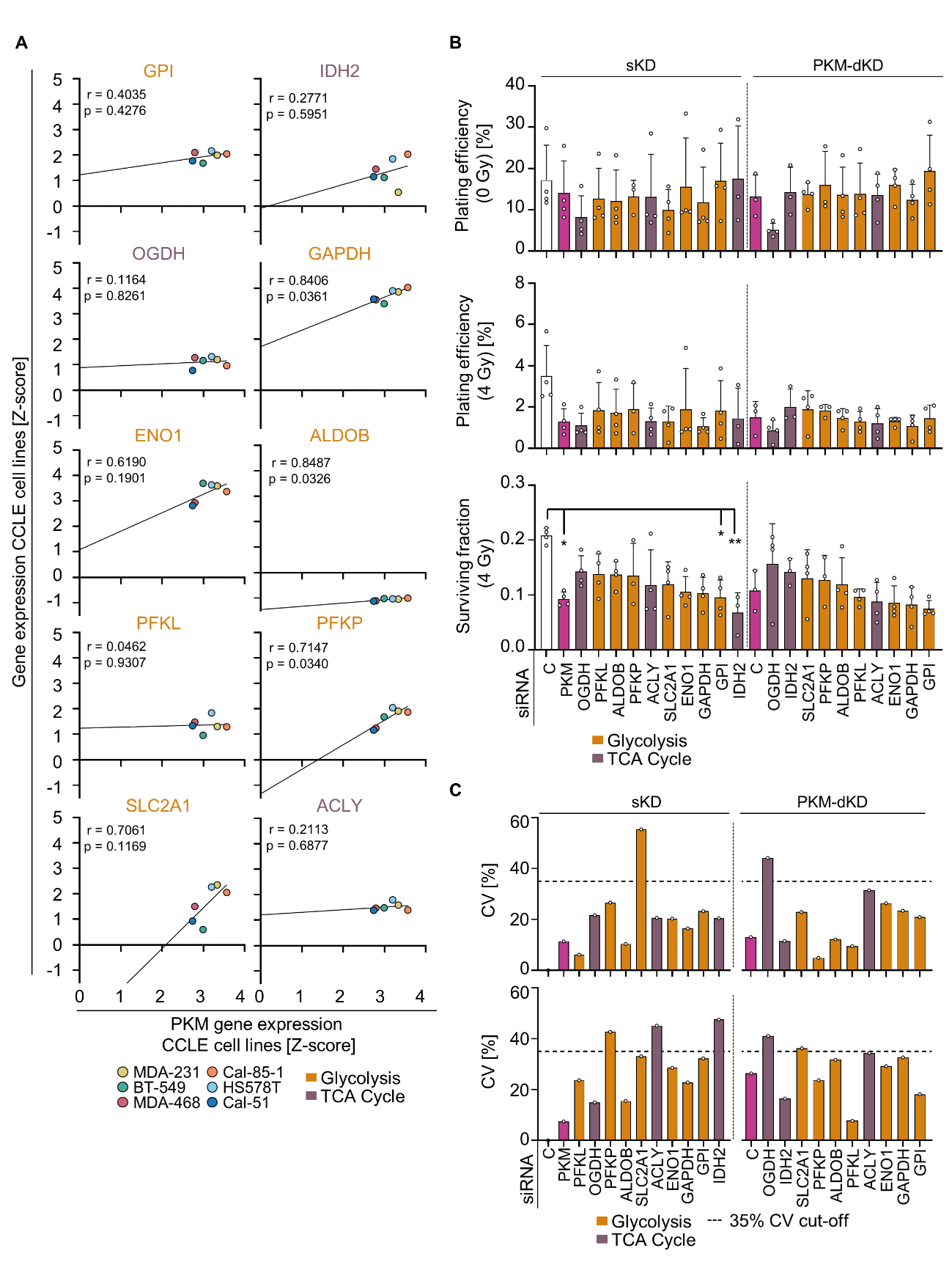
**

**Supplementary Fig. S9. Role of glycolysis and TCA cycle enzymes in PKM-mediated survival and radiation response in TNBC. (A)** Correlation of gene expression between PKM and the selected genes in ‘**Fig 6C’** in the indicated TNBC cell models. RNA-seq data were obtained from CCLE, downloaded from DepMap, and presented as Z-score. Pearson correlation coefficient (r) and p-values were obtained from Prism. **(B)** Platting efficiencies from non-irradiated and irradiated MDA-MB-231 cells, and the corresponding 4 Gy survival fraction for single and PKM-double knockdown of the indicated enzymes. Data n ≥ 3 are presented as mean ± SD and analyzed using one-way ANOVA and indicated as: * p < 0.05; ** p < 0.01. **(C)** Coefficient variance (CV) from data in ‘**Fig. 6F’**, calculated using Microsoft Excel. CV threshold was established at the indicated cut-off of 35 %.

**
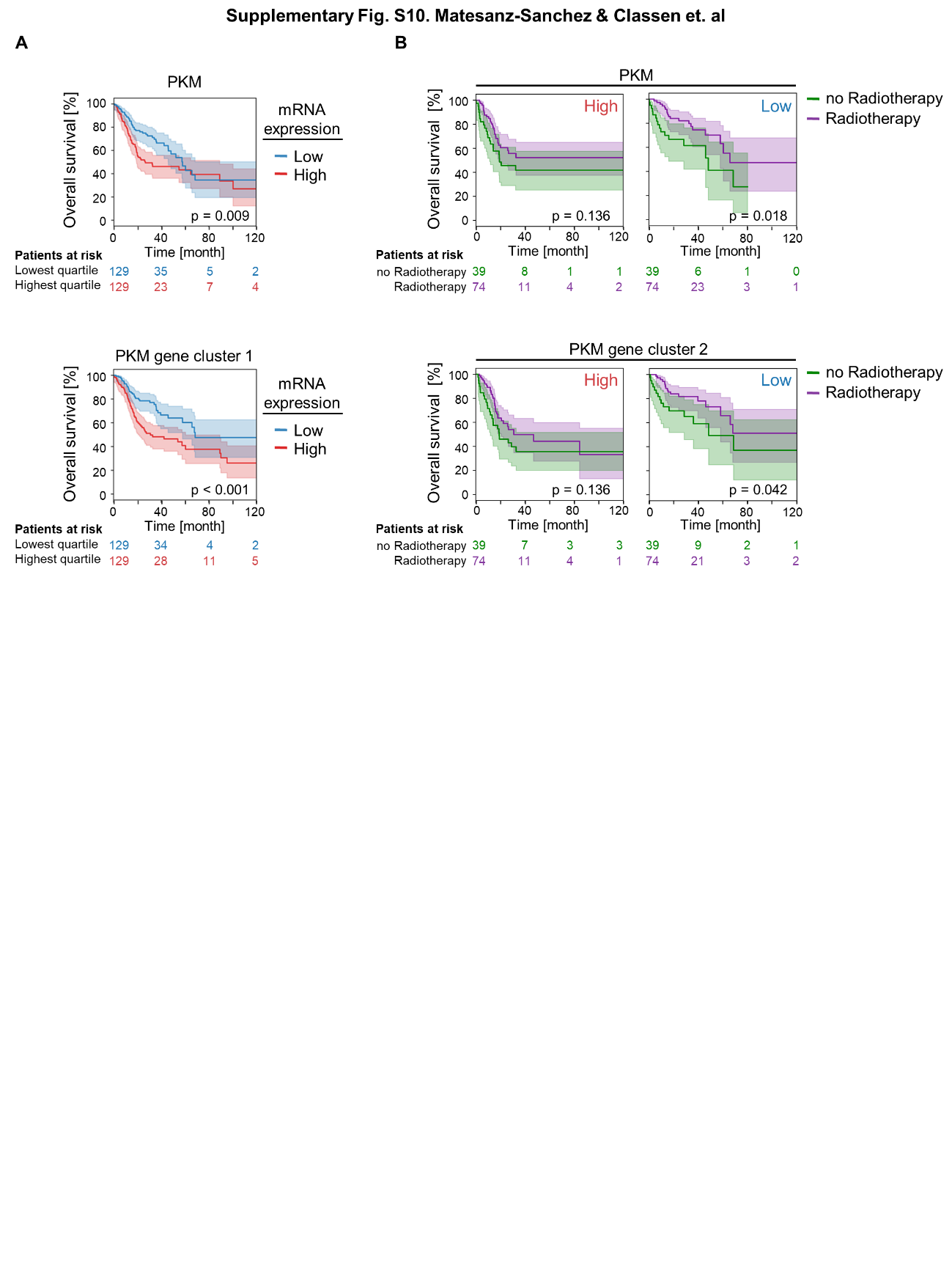
**

**Supplementary Fig. S10. Impact of PKM and PKM-associated gene clusters on survival in Head and Neck Squamous Cell Carcinoma patients. (A)** Kaplan-Meier analyses of overall survival (OS) based on mRNA expression of PKM and PKM-associated gene cluster 1 (from ‘**Fig. 6F**’), comparing the highest vs. the lowest mRNA expression quartiles. **(B)** Kaplan-Meier analysis of Head and Neck Squamous Cell Carcinoma patients to evaluate the impact of RT on OS in high and low expression of PKM and PKM-associated cluster 2 (from **Fig. 6F**’). Data in **A** and **B** were derived from the TCGA PanCancer Atlas via CbioPortal.org. Ten-year OS curves include the confidence intervals, log-rank test p-values, and the patients at risk.
